# The genome sequence of star fruit (*Averrhoa carambola*)

**DOI:** 10.1038/s41438-020-0307-3

**Published:** 2020-06-01

**Authors:** Shasha Wu, Wei Sun, Zhichao Xu, Junwen Zhai, Xiaoping Li, Chengru Li, Diyang Zhang, Xiaoqian Wu, Liming Shen, Junhao Chen, Hui Ren, Xiaoyu Dai, Zhongwu Dai, Yamei Zhao, Lei Chen, Mengxia Cao, Xinyu Xie, Xuedie Liu, Donghui Peng, Jianwen Dong, Yu-Yun Hsiao, Shi-lin Chen, Wen-Chieh Tsai, Siren Lan, Zhong-Jian Liu

**Affiliations:** 1 Key Laboratory of National Forestry and Grassland Administration for Orchid Conservation and Utilization at College of Landscape Architecture, Fujian Agriculture and Forestry University, Fuzhou, 350002 China; 2 Institute of Chinese Materia Medica, Chinese Academy of China Medical Sciences, Beijing, 100700 China; 3 Institute of Medicinal Plant Development, Chinese Academy of Medical Sciences & Peking Union Medical College, Beijing, 100193 China; 4 State Key Laboratory of Subtropical Silviculture, Zhejiang A&F University, Lin’an, Hangzhou, 311300 China; 5 Horticulture Research Institute, Guangxi Academy of Agricultural Sciences, Nanning, 530007 China; 6 Orchid Research and Development Center, National Cheng Kung University, Tainan, 701 China; 7 Institute of Tropical Plant Sciences and Microbiology, National Cheng Kung University, Tainan City, 701 China

**Keywords:** Evolutionary biology, Evolution, Genome

## Abstract

Oxalidaceae is one of the most important plant families in horticulture, and its key commercially relevant genus, *Averrhoa*, has diverse growth habits and fruit types. Here, we describe the assembly of a high-quality chromosome-scale genome sequence for *Averrhoa carambola* (star fruit). *Ks* distribution analysis showed that *A. carambola* underwent a whole-genome triplication event, i.e., the gamma event shared by most eudicots. Comparisons between *A. carambola* and other angiosperms also permitted the generation of Oxalidaceae gene annotations. We identified unique gene families and analyzed gene family expansion and contraction. This analysis revealed significant changes in MADS-box gene family content, which might be related to the cauliflory of *A. carambola*. In addition, we identified and analyzed a total of 204 nucleotide-binding site, leucine-rich repeat receptor (NLR) genes and 58 WRKY genes in the genome, which may be related to the defense response. Our results provide insights into the origin, evolution and diversification of star fruit.

## Introduction

Wood sorrel (Oxalidaceae family) includes approximately 780 species and is distributed in both tropical and temperate areas. It contains species with various forms, including herbs, shrubs, and trees^1^. Wood sorrels are important economic crops and are utilized for both ornamental decoration and medicinal applications^2,3^. Based on morphological and molecular data, Oxalidaceae belongs to Oxalidales and is sister to the Connaraceae family. It can be divided into two main subfamilies: Oxalidoideae and Averrhooideae^4^. Averrhooideae differs from Oxalidoideae, an herbaceous subfamily, by having woody plants. It has four genera and is classified into two tribes: Biophyteae (*Biophytum*) and Averrhoeae (*Dapania*, *Averrhoa* and *Sarcotheca*). It is mostly distributed in tropical and subtropical regions^5^. Although these genera share synapomorphies with other wood sorrels, such as floral morphology, *Averrhoa* possesses several unique traits, including imparipinnate leaves, an herbaceous to papyraceous form, lateral petiolules that do not leave a stalk on the rachis after dropping, and the presence of 3–7 ovules per locule^1^. Therefore, *Averrhoa* is a key taxon in the evolutionary assessment of wood sorrel structure, and analysis of its genome should reveal new insights into the key adaptations that contribute to the diversification within Oxalidaceae^6,7^.

*Averrhoa carambola*, known as star fruit, originated in Asia and has been cultivated in Southeast Asia and Malaysia for many centuries^8–10^. Because the flesh is juicy and rich in vitamin C, star fruit is a commonly consumed tropical fruit^11^. In China, the total consumption of star fruit is approximately 2.6 million tons per year, while the annual production of star fruit in China is approximately two million tons^11^. In addition, star fruit is widely cultivated as a street tree in southern Chinese cities due to its dense foliage and star-like fruit^12^. Furthermore, the characteristics of bearing flowers and fruits on the main trunk and flowering year-round when temperatures exceed 27 °C in tropical regions^13^ make it an ideal species with which to explore economic and interesting traits (cauliflory, defined as flowering from the lower branch and trunk areas of woody plants, and high yield) at the whole-genome scale.

*Cephalotus follicularis* is a carnivorous plant native to southwest Australia that belongs to the monospecific family Cephalotaceae and is the only species of the order Oxalidales with a sequenced and annotated genome^14^. The relationship between star fruit and *Cephalotus follicularis* is not currently known and could be better understood through a comparison of their sequenced genomes.

Here, we present a complete genome sequence for *A. carambola*. Comparisons of genomic data with those from other flowering plants provide fundamental insights into the origin, evolution, adaptation, and diversification of star fruit.

## Results and discussion

### Genome sequencing and characterization

*A. carambola* has a karyotype of 2*N* = 2X = 22 chromosomes^15^. To sequence its genome, we utilized Illumina HiSeq short reads. We obtained a total of 131 Gb of raw reads with short inserts after library construction and sequencing (Supplementary Table 1). The *A. carambola* genome was estimated to be 357.79 Mb in size with a heterozygosity of 1.15% based on 17-*K*-*mer* analysis (Supplementary Fig. 1). Next, we utilized Oxford Nanopore Technology (ONT) and obtained a total of 52.33 Gb of long reads (Supplementary Table 1). The ONT reads were corrected and assembled to produce a 335.49 Mb genome with a contig N50 size of 4.22 Mb (Supplementary Table 2). Then, the draft assembly was polished using short reads, and BUSCO (Benchmarking Universal Single-Copy Orthologs, v3.1.0) assessment indicated that the completeness of the genome was 96.30%, suggesting that the *A. carambola* genome is nearly complete and of high quality (Supplementary Tables 2, 3).

We additionally used 42.76 Gb of Hi-C clean data to reconstruct physical maps by reordering and clustering the assembled scaffolds. We anchored 90.88% of the assembly (305.13 Mb) onto 11 pseudochromosomes using a hierarchical clustering strategy (Supplementary Table 4). Chromatin interaction data were used to assess the quality of the Hi-C assembly, which indicated that the assembly was of high quality (Supplementary Fig. 2). The length of the pseudochromosomes ranged from 17.89 Mb to 33.98 Mb with an N50 value of 31.25 Mb (Supplementary Tables 4, 5).

Approximately 61.3% of the *A. carambola* genome was found to be composed of repetitive elements (transposon elements, TEs) (Supplementary Figs. 3, 4 and Supplementary Table 6). We confidently annotated 25,419 protein-coding genes (Supplementary Table 7 and Supplementary Fig. 5), of which 21,316 (83.86%) were supported by transcriptome data (Supplementary Table 8). A total of 94.8% of annotated BUSCO gene models were identified, suggesting the near completeness of gene prediction (Supplementary Table 8). In addition, we identified 86 microRNAs, 581 transfer RNAs, 71 ribosomal RNAs and 212 small nuclear RNAs in the *A. carambola* genome (Supplementary Table 9).

### Evolution of gene families

**Fig. 1:**
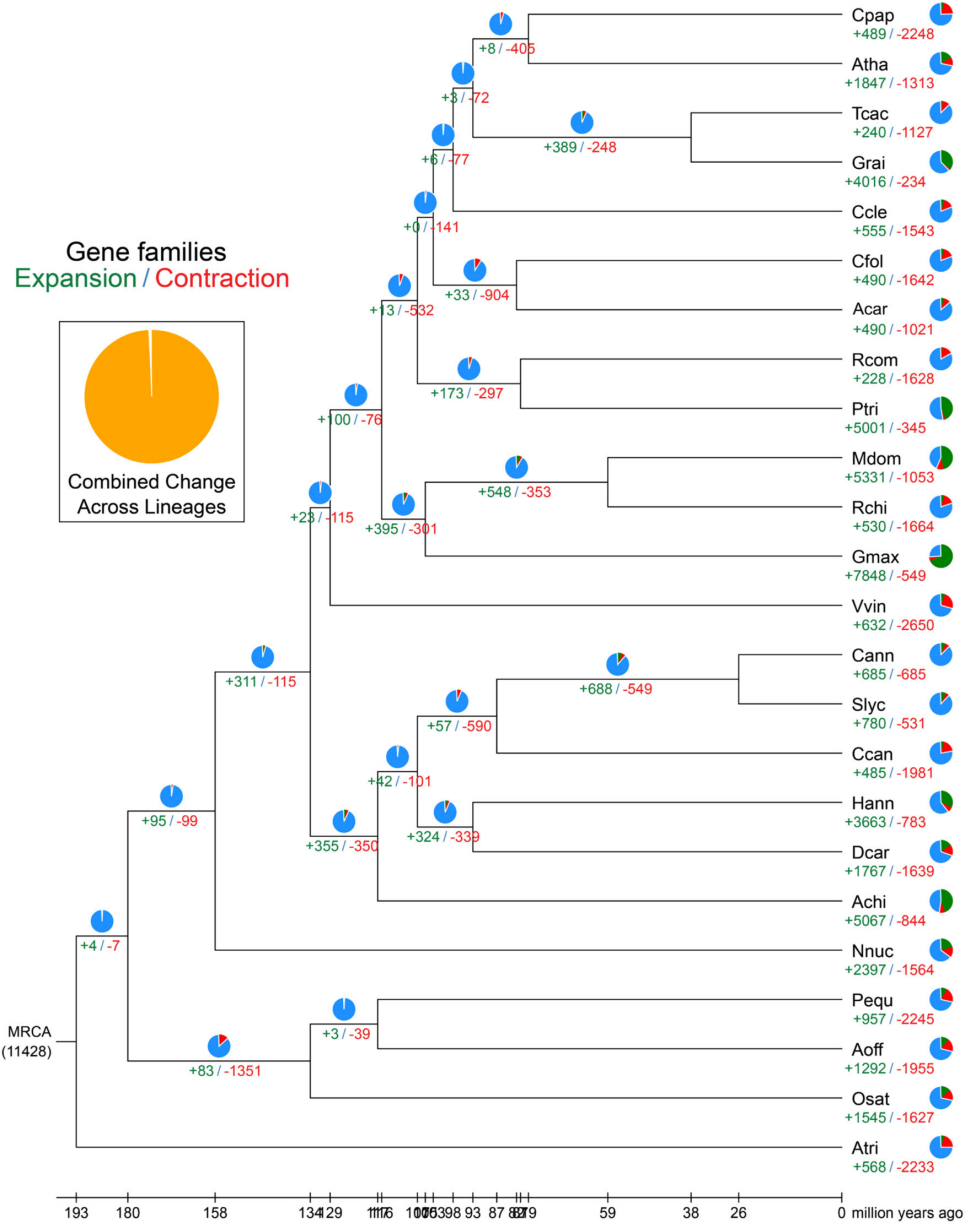
The expansion and contraction of gene families. The green number indicates the number of expanded gene families, while the red number indicates the number of contracted gene families.

We then constructed a high-confidence phylogenetic tree and estimated the divergence times of 24 different plant species based on genes extracted from a total of 93 single-copy families (see Methods and Supplementary Table 10). The estimated divergence time of this set of Oxalidaceae species was 102 Mya (Supplementary Fig. 6). Next, we determined the expansion and contraction of orthologous gene families using CAFÉ 4.2 (Supplementary Fig. 7). Thirty-three gene families were expanded in the lineage leading to Oxalidales, whereas 904 families were contracted (Fig. 1). Four hundred ninety gene families were expanded in *A. carambola*, while 1021 gene families were contracted (Fig. 1).

By comparing 24 different plant species, 504 gene families, including 8153 *A. carambola* genes, appeared to be unique to carambola (see Methods, Supplementary Figs. 7, 8 and Supplementary Table 10). We performed GO and KEGG enrichment analysis (Supplementary Table 11) and found that these gene families were enriched in several categories (Supplementary Tables 12–17).

### Collinearity analysis

**Fig. 2:**
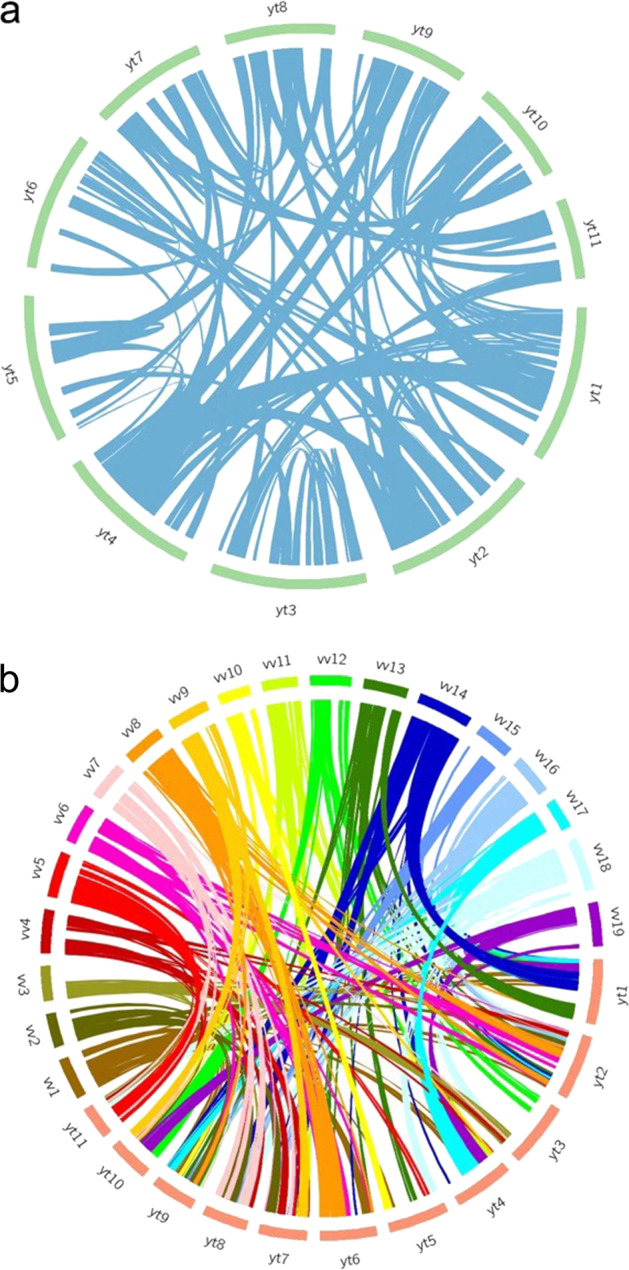
Collinear analysis. (**a**) Circos collinearity plot of *A. carambola*. (**b**) Circos collinearity plot of *A. carambola* with *Vitis vinifera.*

Genes are typically conserved both in function and order inside collinear fragments among closely related species. We utilized MCScanX^16^ to assess the collinearity among species related to *Averrhoa carambola* and found that all the predicted genes except TE-related genes were highly conserved in both function and order (Fig. 2 and Supplementary Figs. 9, 10).

### Whole-genome duplication

Whole-genome duplication (WGD) is a process of genome doubling that dramatically increases genome complexity. One of the striking features of plant genomes is that WGD has occurred many times^17,18^. WGD is a particularly important feature of angiosperm genomes. To determine whether the *Averrhoa* genome had undergone WGD during evolution, we used *Ks* (synonymous substitutions per site) distribution analysis. The paralogous gene pairs were extracted from the OrthoMCL results, and the *Ks* values were calculated using CodeML in the PAML package^16,19^.

**Fig. 3:**
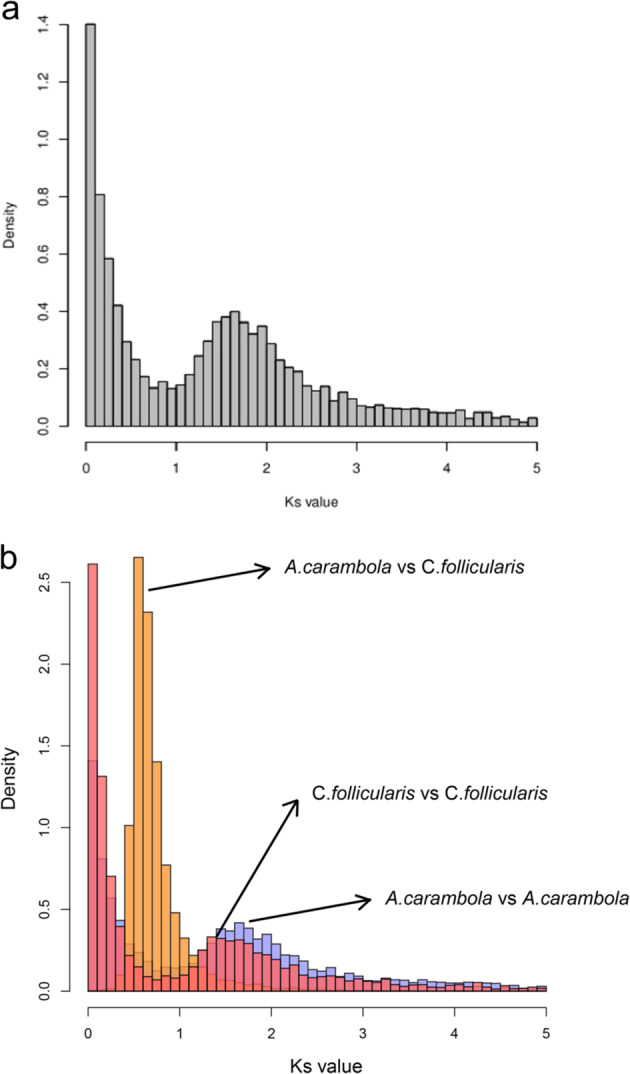
*Ks* distribution of *Averrhoa carambola*. (**a**) *Ks* distribution of *A. carambola* paralogous genes. (**b**) *Ks* distribution of *A. carambola* and *Cephalotus follicularis.*

There was a peak between *Ks* values of 1.6–1.8, indicating that the *A. carambola* genome had undergone a WGD event. Further analysis of *A. carambola* and *C. follicularis* revealed that the common ancestor prior to the differentiation of *Averrhoa* and *C. follicularis* experienced a WGD event. This event was likely the γ event shared by most core eudicots (Fig. 3). Comparative genomic analysis between the *A. carambola* and *C. follicularis* genomes revealed a one-to-one syntenic relationship, suggesting that no WGD events occurred after speciation (Supplementary Fig. 11).

### MADS-box genes of star fruit

MADS-box genes are known to be involved in many important processes during plant development and are especially known for their roles in flowering and flower development^20^. Because *Averrhoa* is well known for its cauliflory, defined as flowering from the lower branch and trunk areas of woody plants, we focused on identifying and characterizing the MADS-box genes in the *Averrhoa* genome in more detail.

**Fig. 4:**
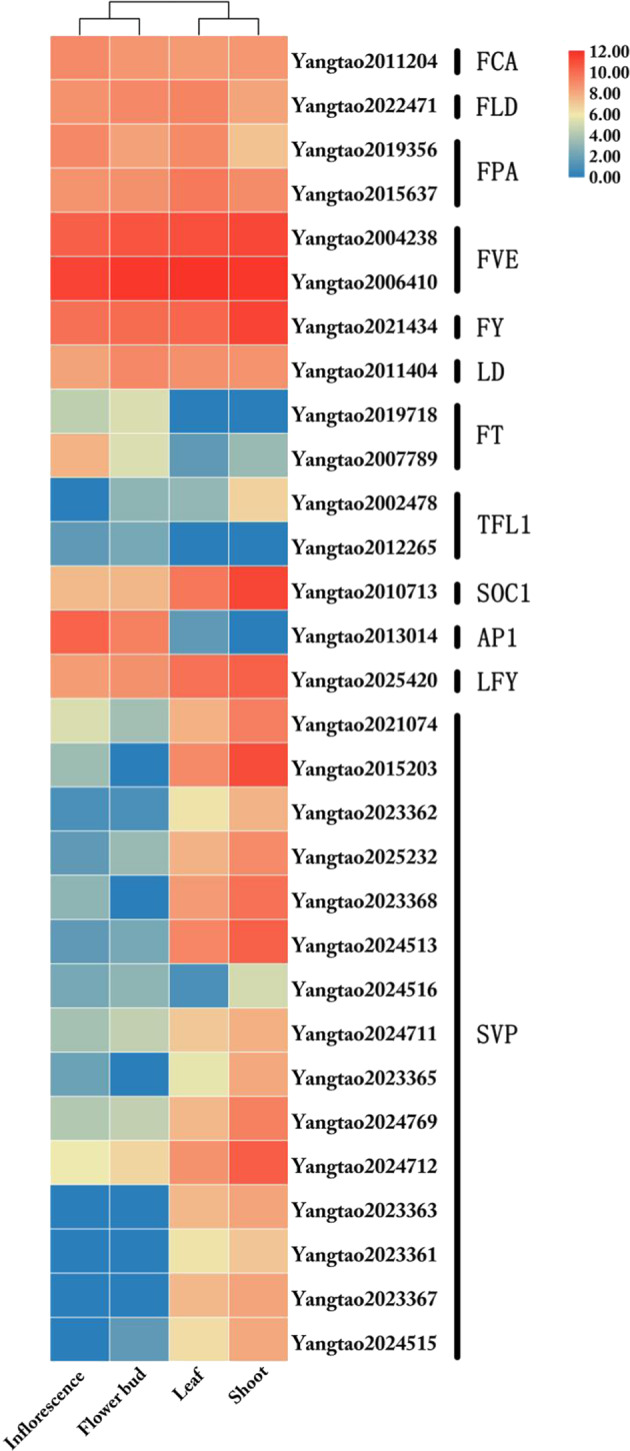
Expression patterns of genes involved in the flowering. Pathway of *Averrhoa carambola.*

In total, 74 putative functional MADS-box genes and two pseudogenes were identified in *A. carambola* (Table 1). This number is less than the number of MADS-box genes found in *Arabidopsis thaliana*^21^ and *Theobroma cacao*^22,23^ but greater than the number found in *C. follicularis* (Table 1). *A. carambola* has 48 type-II MADS-box genes, which is comparable to the number in *A. thaliana* (45) but less than the number found in *T. cacao* (67) (Table 1). Phylogenetic analysis (Supplementary Fig. 12) showed that many of the type-II MADS-box genes were duplicated, except those in the B-class, PI, FLC, AGL12, and Bs clades. The duplicated type-II clades included A-class (three members), B-class AP3 (two members), C/D-class (three members), E-class (four members), AGL6 (two members), SOC1 (three members), AGL15 (two members), ANR1 (five members), SVP (15 members), and MICK* (five members) clades (Table 1). Notably, we found that the SVP (15 members) clade in *A. carambola* contained more genes than that in *A. thaliana*, *C. follicularis* and cocoa tree (Table 1). The large number of SVP clade members might be due to tandem duplication. In *Arabidopsis*, the two *SVP* paralogs *SVP* and *AGL24* are involved in floral transition and development. *SVP* suppresses flowering by acting with *FLC* to negatively regulate *SOC1* and *FT*^24^. In contrast, *AGL24* acts as a flowering activator to activate *SOC1* expression during inflorescence development^25^. We detected transcripts of all *SVP*-like genes except *Yangtao2024516* in vegetative leaves and shoots (Fig. 4). Interestingly, expression of an *SVP*-like gene (*Yangtao2024516*) was detected in the inflorescence and flower buds (Fig. 4). These results suggested that *Yangtao2024516* functions in flowering activation, and the other 14 *SVP*-like genes might also be related to flowering suppression. The expanded *SVP* clade, including members with differential expression patterns in *A. carambola*, might contribute to flowering regulatory networks and relate to *A. carambola* cauliflory.

**Table 1:** MADS-box genes in *Averrhoa carambola, Arabidopsis thaliana*, *Cephalotus follicularis* and *Theobroma cacao.*

Category	*A. carambola*		*A. thaliana*^21^		*C. follicularis*^14^		*T. cacao*^22,23^	
	Functional	Pseudo	Functional	Pseudo	Functional	Pseudo	Functional	Pseudo
Type II (Total)	48^a^		45		27		68	
MIKC^c^	43		39		24		58	
A	3		4		3		7	
AGL12	1		1		1		1	
C/D	3		4		3		10	
SOC1	3		6		3		7	
SVP	15		2		2		4	
ANR1	5		4		2		3	
Bs	1		2		1		4	
B-PI	1		1		1		1	
B-AP3	2		1		2		5	
AGL6	2		2		1		5	
E	4		4		3		6	
FLC	1		6		0		3	
AGL15	2		2		2		2	
MIKC^a^	5		6		3		10	2
Type I (Total)	28	2	61		24		28	
Mα	20		25		15		8	3
Mβ	2	2	20		5	2	2	1
Mγ	4		16		4		13	1
Total	74	2	106		51	2	91	7

^a^The number of MADS-box genes identified in this study. Genes with a stop codon in the MADS-box domain were categorized as pseudogenes.

**Fig. 5:**
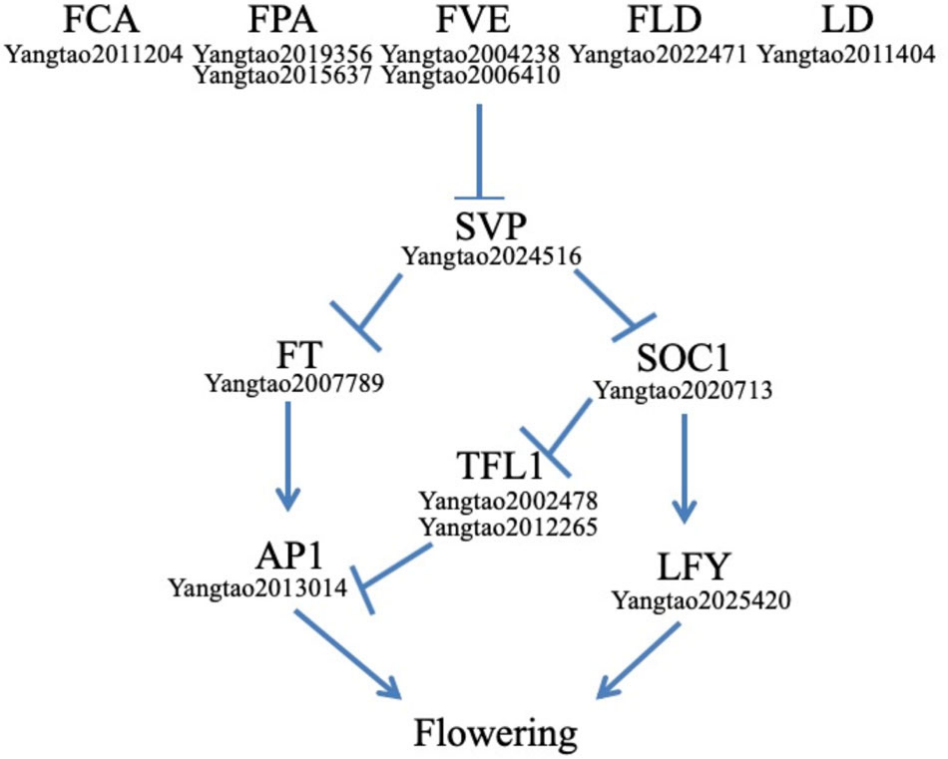
Putative autonomous flowering. Time pathway in *Averrhoa carambola.*

Cauliflorous flowering occurs due to the presence of adventitious buds that remain dormant and take years to develop after their formation, when the trunk develops and thickens due to secondary growth^26^. It has been reported that *SVP2* functions in the suppression of meristem activity to prevent precocious budbreak in the perennial species kiwifruit^27^. The flowering repressor *SVP* is also controlled by the autonomous pathway and directly represses *SOC1* transcription in *Arabidopsis*^28^. Interestingly, we found that genes involved in the autonomous flowering time pathway might repress the expression of *SVP*-like genes in reproductive tissues to promote flowering through activation of the floral integrator genes *FLOWERING LOCUS T* and *SOC1* (Figs. 4, 5). Further study on the *A. carambola* flowering time pathways will improve our knowledge about causal genes, with applications in commercial variety selection.

Compared with type-II MADS-box genes, only 26 putative functional type-I genes and two pseudogenes were characterized (Table 1), which may have been due to the low duplication rate and high loss rate of type-I MADS-box genes. Tandem duplications seem to have contributed to more type-I genes, especially those in the Mα group. This suggests that type-I MADS-box genes have mainly been duplicated during smaller-scale and more-recent duplications^21^. Functional studies of type-I MADS-box genes in *A. carambola* will advance our understanding of their role in Oxalidaceae.

### Analysis of NLR genes and the WRKY gene family

Resistance (R) genes produce R proteins to provide plant disease resistance against pathogens, most of which are nucleotide-binding site leucine-rich repeat (NLR) genes. NLRs contain three classes based on their N-terminal domain structures, namely, the toll/interleukin-1 receptor (TIR) NLR (TNL), coiled-coil (CC) NLR (CNL), and resistance to powdery mildew8 (RPW8) NLR (RLN)^29^. Here, a genome-wide identification of NLR genes in star fruit was carried out, resulting in the identification of 204 NLR genes, including homologues of known resistance genes: RNL (with 13 members), TNL (with 41 members) and CNL (with 150 members) (Table 2). There seem to be more NLR genes in *A. carambola* than in *A. thaliana* (174 genes)^30^ but fewer than in the basal angiosperm *Nymphaea colorata* (342 genes)^31^. Phylogenetic analysis of the NLR genes indicated that tandem duplication contributed to the increasing number of CNL genes. Remarkably, the NLR genes in the *C. follicularis* genome^14^ showed sharp contraction after the speciation of *A. carambola* and *C. follicularis*, with only one RNL and one TNL identified (Supplementary Fig. 13).

**Table 2:** NLR genes in *Averrhoa carambola, Arabidopsis thaliana*, *Cephalotus follicularis* and *Nymphaea colorata.*

	*A. carambola*	*A. thaliana*^30^	*C. follicularis*^14^	*N. colorata*^31^
RNL	13	9	1	22
TNL	41	101	1	57
CNL	150	64	26	263
total	204	174	28	342

*RNL* RPW8-NLR, *TNL* TIR-NLR, *CNL* CC-NLR, *NLR* nucleotide-binding site leucine-rich repeat, *RPW8* resistance to powdery mildew 8, *TIR* toll/interleukin-1 receptor, *CC* coiled-coil.

Plant WRKY proteins are transcription factors (TFs) involved in regulating plant growth and development, as well as responses to many biotic and abiotic stresses^32^. An investigation of the star fruit genome revealed 58 WRKY genes. There appear to be fewer WRKY genes in star fruit than in the model plant *A. thaliana* (72 genes)^33^ and the basal angiosperm *N. colorata* (69 genes)^31^. The closely related species *C. follicularis* contains 42 WRKY genes^14^ (Table 3). Phylogenetic analysis showed that WRKY genes, especially type-IIc and type-I genes, underwent expansion in *A. carambola*, with the exception of those in the type-IIa clade. Among the 58 WRKY genes, type I (with 10 members), type II (33 members) and type III (eight members) contained more members than in *C. follicularis* (six members in type I, 29 members in type II and seven members in type III) (Supplementary Fig. 14). Interestingly, there was an unknown clade present in *A. thaliana* and *C. follicularis*, which contained three members in the star fruit genome and two members in the *N. colorata* genome.

**Table 3:** WRKY genes in *Averrhoa carambola, Arabidopsis thaliana*, *Cephalotus follicularis* and *Nymphaea colorata.*

	*A. carambola*	*A. thaliana*^32^	*C. follicularis*^14^	*N. colorata*^31^
I	10	13	6	15
IIa	3	3	7	5
IIb	7	8	4	9
IIc	15	17	7	16
IId	6	7	4	8
IIe	6	9	7	7
III	8	14	7	7
Unknown	3	0	0	2
Out	0	1	0	0
Total	58	72	42	69

## Conclusion

Although star fruit is well known as a delicacy in the tropics, research on it has been hampered by the absence of genetic resources. We identified new gene families and analyzed gene family expansion and contraction by comparisons with related species. Significant changes were found in the MADS-box gene family, which might be related to the cauliflory of *A. carambola*. Evolutionary analysis revealed one WGD event, which was the γ event experienced by most eudicots. The *A. carambola* assembly represents the first from the wood sorrel family and thus provides a valuable resource for evolutionary phylogenomic studies.

## Material and methods

### Plant sample preparation and sequencing

Diploid *A. carambola* was cultivated at the South China Botanical Garden, Guangzhou, Guangdong Province, China. Fresh young leaves were collected to extract genomic DNA for 400 bp paired-end library construction and Illumina sequencing. Genome size and heterozygosity were calculated using KmerFreq and GCE based on a 17-*K-mer* distribution. The high-molecular-weight DNA of fresh young leaves was extracted and randomly fragmented to construct a 20 kb library for Oxford Nanopore sequencing.

### Genome assembly and chromosome anchoring

The raw fastq data from ONT sequencing were transformed and filtered using MinKNOW software. Canu (v1.7) was used to correct and trim the raw ONT reads with the default parameters. The corrected and trimmed ONT reads were assembled by SMARTdenovo using 21-mers^34^. The assembled contigs were then polished three times with Pilon (v1.22) using Illumina short reads. The quality of the genome assembly was estimated by searching for BUSCO v3.1.0 using the Embryophyta ODB 10 database.

Fresh leaves of *A. carambola* were used to construct a Hi-C sequencing library, including chromatin crosslinking, chromatin digestion with HindIII, biotin labeling and end repair, DNA purification and streptavidin pull-down of labeled Hi-C ligation products. The library was then sequenced using the Illumina platform, and the clean sequences were mapped to the draft genome, with valid Hi-C reads employed to correct the draft assembly. Finally, the draft genome of *A. carambola* was assembled into chromosomes using Lachesis (9).

### Identification of repetitive sequences

Tandem repeats across the genome were predicted using Tandem Repeats Finder (v4.07b, http://tandem.bu.edu/trf/trf.html). Transposable elements (TEs) were first identified using RepeatMasker (http://www.repeatmasker.org, v3.3.0) and RepeatProteinMask based on the Repbase TE library^35^. Next, two de novo predication software programs, RepeatModeler (http://repeatmasker.org/RepeatModeler.html) and LTR_FINDER^36^, were used to identify TEs in the star fruit genome. Finally, repeat sequences with identities ≥ 50% were grouped into the same class.

### Gene prediction and annotation

Three independent methods were used for gene prediction. Homologous sequence searching was performed by comparing protein sequences of five sequenced species against the star fruit genome using the TBLASTN algorithm with a cut-off E-value of ≤ 1e^−5^. Then, the corresponding homologous genome sequences were aligned against BLAST hits using GeneWise v2.4.1 to extract accurate exon–intron information^37^. Three *ab initio* prediction software programs, Augustus v3.0.2^38^, FGENESH^39^ and GlimmerHMM^40^, were employed for de novo gene prediction. Then, the homology-based and *ab initio* gene structures were merged into a nonredundant gene model using GLEAN. The completeness of gene prediction was evaluated using BUSCO v3.1.0. Finally, the RNA sequencing (RNA-Seq) reads were mapped to the assembly using TopHat v2.0.11^41^, and Cufflinks v2.2.1^42^ was applied to combine mapping results for transcript structural predictions.

The protein sequences of the consensus gene set were aligned to various protein databases, including GO (The Gene Ontology Consortium), KEGG^43^, InterPro^44^, Swiss-Prot and TrEMBL, for the annotation of predicted genes. The rRNAs were identified by aligning the rRNA template sequences from the Rfam^45^ database against the genome using the BLASTN algorithm at an E-value cut-off of 1e^−5^. The tRNAs were predicted using tRNAscan-SE^46^, and other ncRNAs were predicted by running Infernal 0.81 software against the Rfam database.

### Genome evolution analysis

Gene families present in the 24 genomes were identified using OrthoMCL^47^. Peptide sequences from 93 single-copy gene families were used to construct phylogenetic relationships and estimate divergence times. Alignments from MUSCLE were then converted to coding sequences. Fourfold degenerate sites were concatenated and used to estimate the neutral substitution rate per year and the divergence time. PhyML^48^ was used to construct a phylogenetic tree. The Bayesian relaxed molecular clock approach was used to estimate species divergence times using the program MCMCTREE v4.0, which is part of the PAML package^49^. The ‘correlated molecular clock’ and ‘JC69’ models were used. Published *Arabidopsis*–*Papaya* (54–90 Mya), *P. trichocarpa*–*Arabidopsis* (100–129 Mya), monocot–dicot (140 Mya) and angiosperm (<200 Mya) divergence times were used for calibration^50^.

### Transcriptome sequencing and expression analysis

The inflorescence, floral bud, leaf and shoot were collected independently from *A. carambola*. Total RNA was extracted according to the manufacturer’s protocol. Illumina RNA-Seq libraries were prepared and sequenced on a HiSeq 2500 system following the manufacturer’s instructions (Illumina, USA). Two biological replicates were analyzed for each sample. To estimate gene expression levels, clean reads of each sample were mapped onto the assembled genome to obtain read counts for each gene using HTSeq-count and normalized to FPKM counts^42^.

### Phylogenetic reconstruction of the MADS-box gene family

For the identification of MADS-box family members in *A. carambola*, BLASTp was applied using known plant MADS-box proteins as reference sequences^21,51^, with an E-value cut-off of ≤ 1e−5. For phylogenetic analyses, a total of 333 protein sequences, including 76 *A. carambola* AcMADS-box, 106 *A. thaliana* AtMADS-box^21^, 53 *C. follicularis* CfMADS-box^14^ and 98 *T. cacao* TcMADS-box^22,23^, were aligned using MUSCLE^52^ (v3.8.31; http://www.drive5.com/muscle) with default parameters. A phylogenetic tree was drawn through RAxML^53^ with the GTRGAMMA substitution model and 1000 bootstraps on the CIPRES website (https://www.phylo.org/portal2/home.action)^54^. The phylogenetic tree was visualized using FigTree software (http://tree.bio.ed.ac.uk/software/figtree) (Supplementary Fig. 12).

### NLR genes and WRKY TF identification

All the annotated protein sequences of *Arabidopsis*, *C. follicularis*, and *N. colorata* were downloaded. The NLR gene (PF00931) and WRKY TF (PF03106) models were obtained from the Pfam database (http://pfam.xfam.org/). The NLR genes and WRKY TFs were then identified using HMMER v3.2.1. The identified sequences were confirmed and filtered using the UniProt database (https://www.uniprot.org/) and the NCBI database (https://www.ncbi.nlm.nih.gov/). Then, MAFFT v7.407 was used to align multiple sequences of candidate proteins with default parameters. A maximum likelihood (ML) phylogenetic tree was constructed for the protein sequences of NLR genes and WRKY TFs from *Arabidopsis*^30,33^, *C. follicularis*^14^, *N. colorata*^31^ and star fruit by FastTree with default parameters.

## Supplementary information

Supplementary Information

## Data Availability

The whole-genome sequence data reported in this paper have been deposited in the Genome Warehouse of the National Genomics Data Center, Beijing Institute of Genomics (BIG), Chinese Academy of Sciences, under accession number GWHABKE00000000 and are publicly accessible at https://bigd.big.ac.cn/gwh.
