# Supplementary material for: The genome sequence of star fruit (Averrhoa carambola)
Source: Hortic Res. 2020 Jun 1;7:95. doi: 10.1038/s41438-020-0307-3 (PMC7261771; doi:10.1038/s41438-020-0307-3)
Supplement: Supplementary file 1 — Supplementary Information [file 41438_2020_307_MOESM1_ESM.docx]

Supplementary Information

**The genome sequence of the star fruit *Averrhoa carambola***

Shasha Wu^1*^, Wei Sun^2*^, Zhichao Xu^3*^, Junwen Zhai^1*^, Xiaoping Li^1^, Chengru Li^1^, Diyang Zhang^1^, Xiaoqian Wu^1^, Liming Shen^1^, Junhao Chen^4^, Hui Ren^5^, Zhongwu Dai^1^, Xiaoyu Dai^1^, Yamei Zhao^1^, Lei Chen^1^, Mengxia Cao^1^, Xinyu Xie^1^, Xuedie Liu^1^, Donghui Peng^1^, Jianwen Dong^1^, Yu-Yun Hsiao^6,7^, Shi-lin Chen^2†^, Wen-Chieh Tsai^6, 7†^, Siren Lan^1†^ and Zhong-Jian Liu^1†^

Content

[Supplementary Figures 4](#_Toc29413606)

[**Supplementary Figure 1. Genome size and heterozygosity estimation using 17 K-mer distribution.** 4](#_Toc29413607)

[**Supplementary Figure 2. Intensity signal heat map of Hi-C chromosome interaction.** 5](#_Toc29413608)

[**Supplementary Figure 3. The sequence divergence rate of four different TEs using RepeatMasker annotation.** 6](#_Toc29413609)

[**Supplementary Figure 4. The sequence divergence rate of four different TEs using de novo annotation.** 7](#_Toc29413610)

[**Supplementary Figure 5. Gene structure prediction results statistics.** 8](#_Toc29413611)

[**Supplementary Figure 6. Species differentiation time.** 9](#_Toc29413612)

[**Supplementary Figure 7. Venn diagram shows the number of orthologous genes families in *A. carambola*, *C. clementina*, *A. thaliana* and *C. follicularis*.** 10](#_Toc29413613)

[**Supplementary Figure 8. Orthologous genes in *A. carambola* and other species.** 11](#_Toc29413614)

[**Supplementary Figure 9. Collinear plot of *A. carambola* with *Vitis vinifera*.** 12](#_Toc29413615)

[**Supplementary Figure 10. Collinear plot of *A. carambola*.** 13](#_Toc29413616)

[**Supplementary Figure 11. Genome comparison between *A. carambola* and *C. follicularis* genome.** 14](#_Toc29413617)

[**Supplementary Figure 12. Phylogenetic tree showing the evolutionary relationship between MADS-box genes in the *A. carambola, T. cacao* and *A. thaliana*.** 15](#_Toc29413618)

[**Supplementary Figure 13. Phylogenetic tree showing the evolutionary relationship of *R* genes in the *A. carambola*, *C. follicularis*, *A. thaliana*, and *N. colorata.*** 16](#_Toc29413619)

[**Supplementary Figure 14. Phylogenetic tree showing the evolutionary relationship of WRKY genes in the *A. carambola*, *C. follicularis*, *A. thaliana*, and *N. colorata.*** 17](#_Toc29413620)

[Supplementary Tables 18](#_Toc29413621)

[**Supplementary Table 1. The statistics of sequencing raw data from Illumina and Nanopore platforms.** 18](#_Toc29413622)

[**Supplementary Table 2. The assembled statistics of *A. carambola* genome.** 19](#_Toc29413623)

[**Supplementary Table 3. BUSCO assessment of the *A. carambola* genome.** 20](#_Toc29413624)

[**Supplementary Table 4. The statistic result of Hi-C assembly.** 21](#_Toc29413625)

[**Supplementary Table 5. The length of chromosome by Hi-C assembly.** 22](#_Toc29413626)

[**Supplementary Table 6. The statistic result of repeat sequence of *A. carambola*.** 23](#_Toc29413627)

[**Supplementary Table 7. The prediction of gene structures of the *A. carambola* genome.** 24](#_Toc29413628)

[**Supplementary Table 8. BUSCO assessment of gene prediction of the *A. carambola* genome.** 25](#_Toc29413629)

[**Supplementary Table 9. Statistics on the annotation of Non-coding RNA of the *A. carambola* genome.** 26](#_Toc29413630)

[**Supplementary Table 10. Statistic result of clustered gene families.** 27](#_Toc29413631)

[**Supplementary Table 11. Statistics on the annotation of the *A. carambola* genome.** 28](#_Toc29413632)

[**Supplementary Table 12. GO enrichment of significant expansion of *A. carambola* gene families.** 29](#_Toc29413633)

[**Supplementary Table 13. KEGG pathway enrichment of significant expansion of *A. carambola* gene families.** 30](#_Toc29413634)

[**Supplementary Table 14. GO enrichment of significant contraction of *A. carambola* gene families.** 31](#_Toc29413635)

[**Supplementary Table 15. KEGG pathway enrichment of significant contraction of *A. carambola* gene families.** 32](#_Toc29413636)

[**Supplementary Table 16. GO enrichment of unique genes of *A. carambola.*** 33](#_Toc29413637)

[**Supplementary Table 17. KEGG pathway enrichment of unique genes of *A. carambola.*** 34](#_Toc29413638)

**Supplementary Figures**

**Supplementary Figure 1. Genome size and heterozygosity estimation using 17 K-mer distribution.**

The X-axis is depth; the Y-axis represents the frequency. According to the distribution, we estimate that the genome size of *A. carambola* is approximately 359.79 Mb.


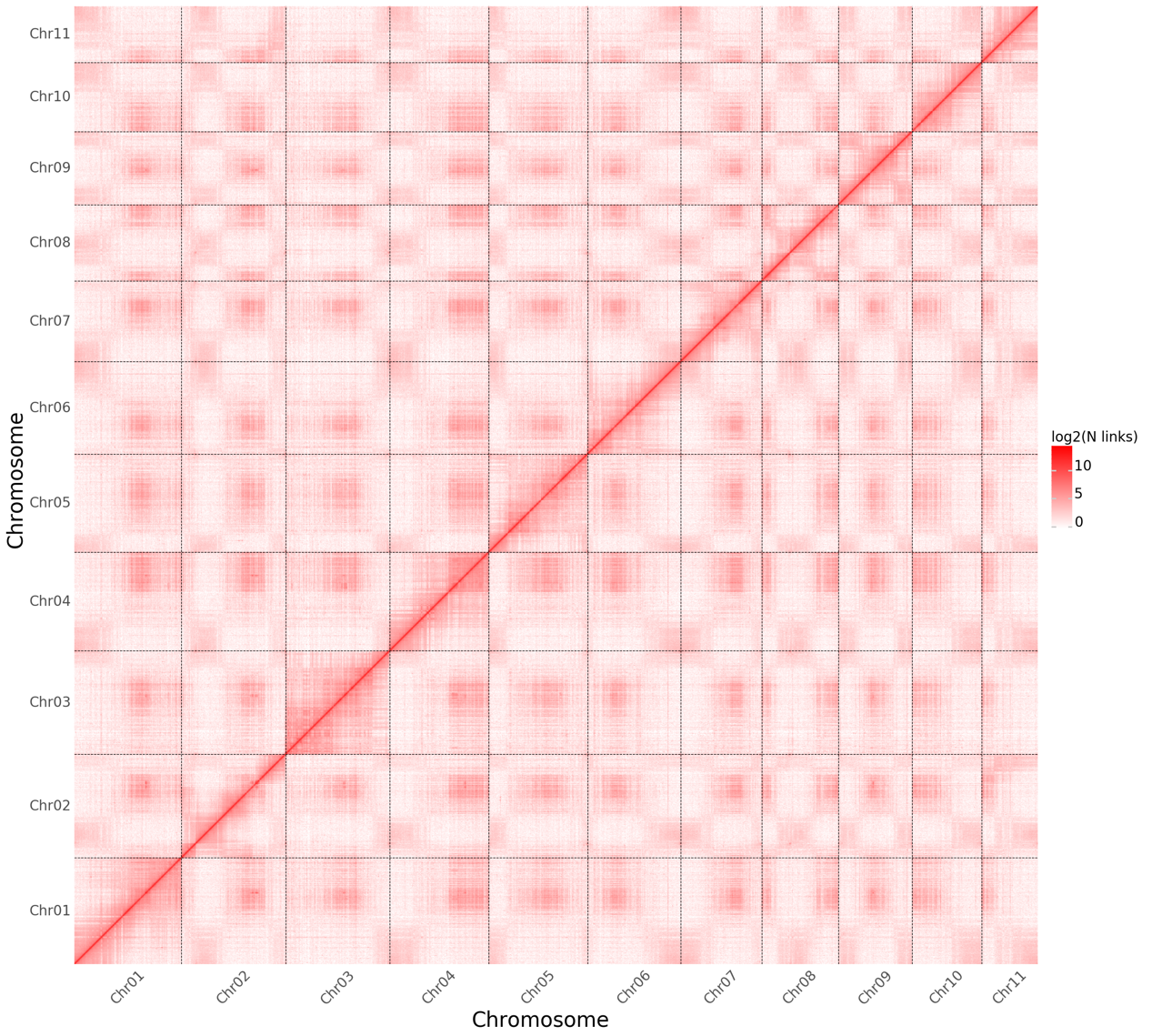


**Supplementary Figure 2. Intensity signal heat map of Hi-C chromosome interaction.**


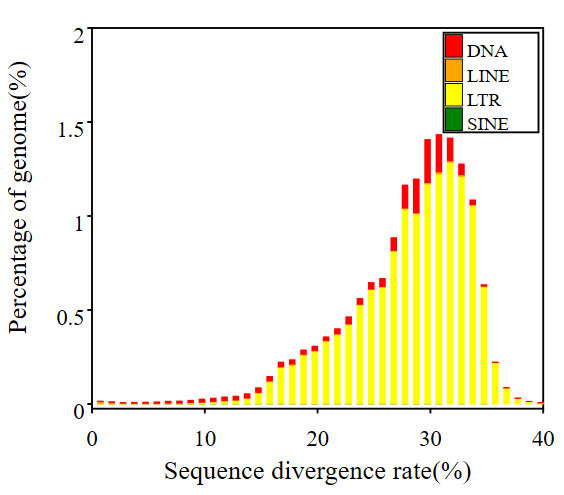


**Supplementary Figure 3. The sequence divergence rate of four different TEs using RepeatMasker annotation.**


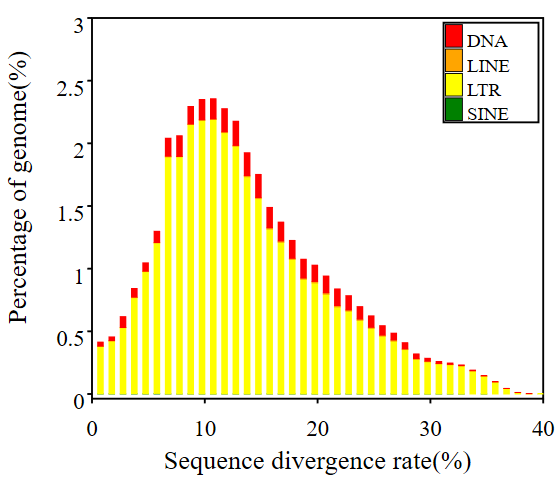


**Supplementary Figure 4. The sequence divergence rate of four different TEs using de novo annotation.**


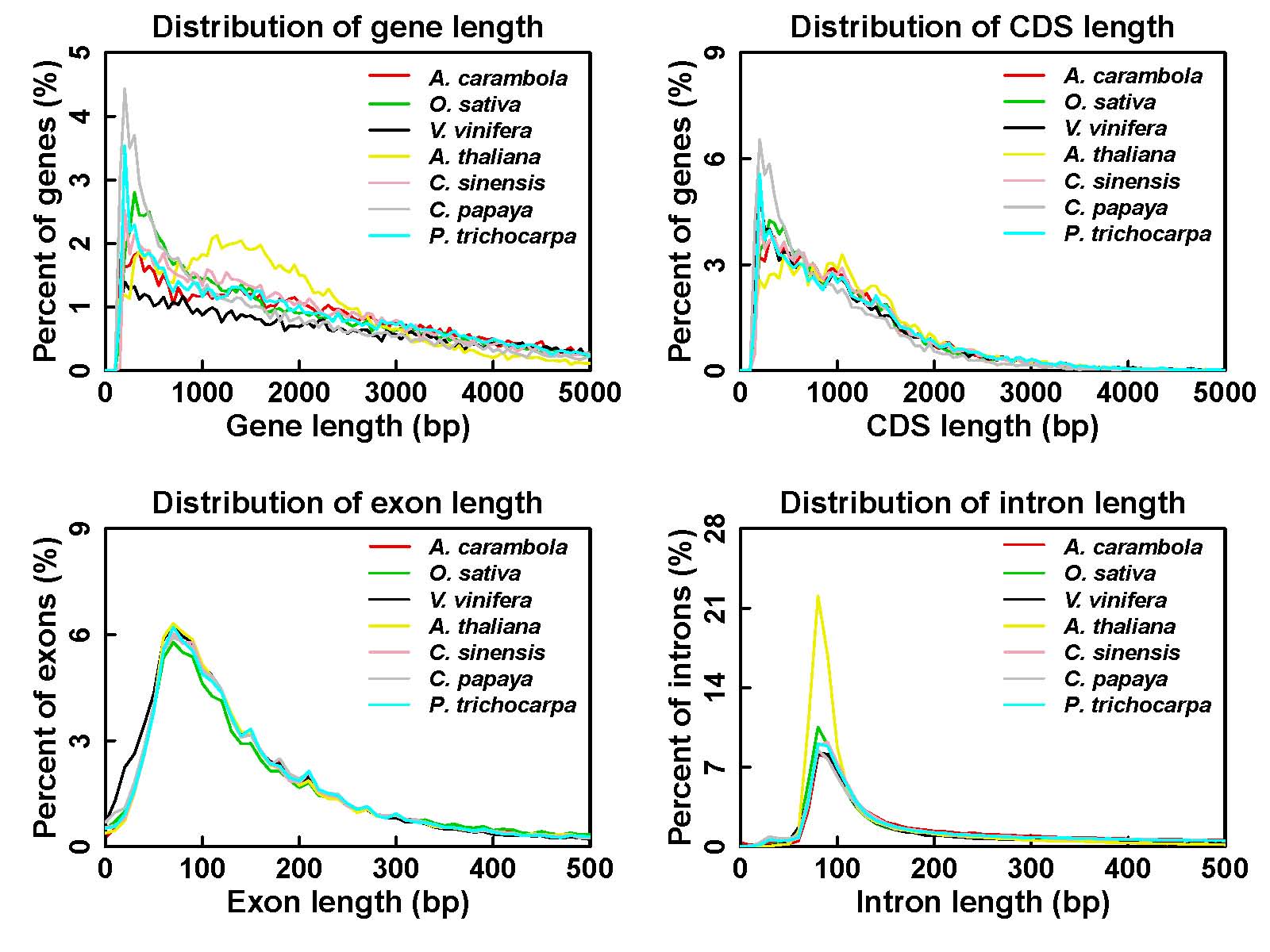


**Supplementary Figure 5. Gene structure prediction results statistics.**

*A. carambola* compared with genetic elements of related species. Window refers to the length represented by each point on the horizontal coordinate.


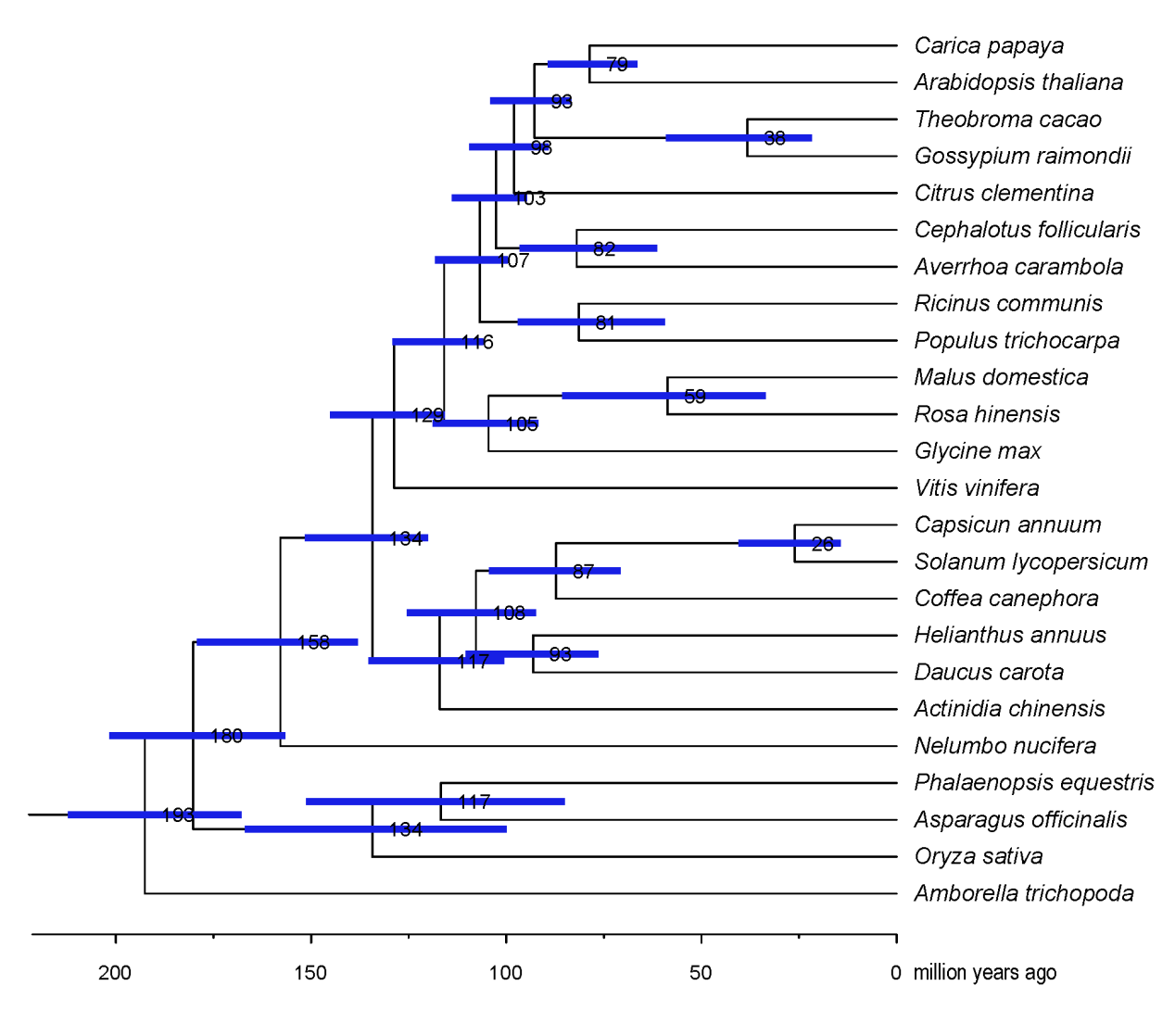


**Supplementary Figure 6. Species differentiation time.**

The purple bar represents the corresponding 95% posterior probability interval for species differentiation estimation, and the geographic time scale unit is million years (Mya).


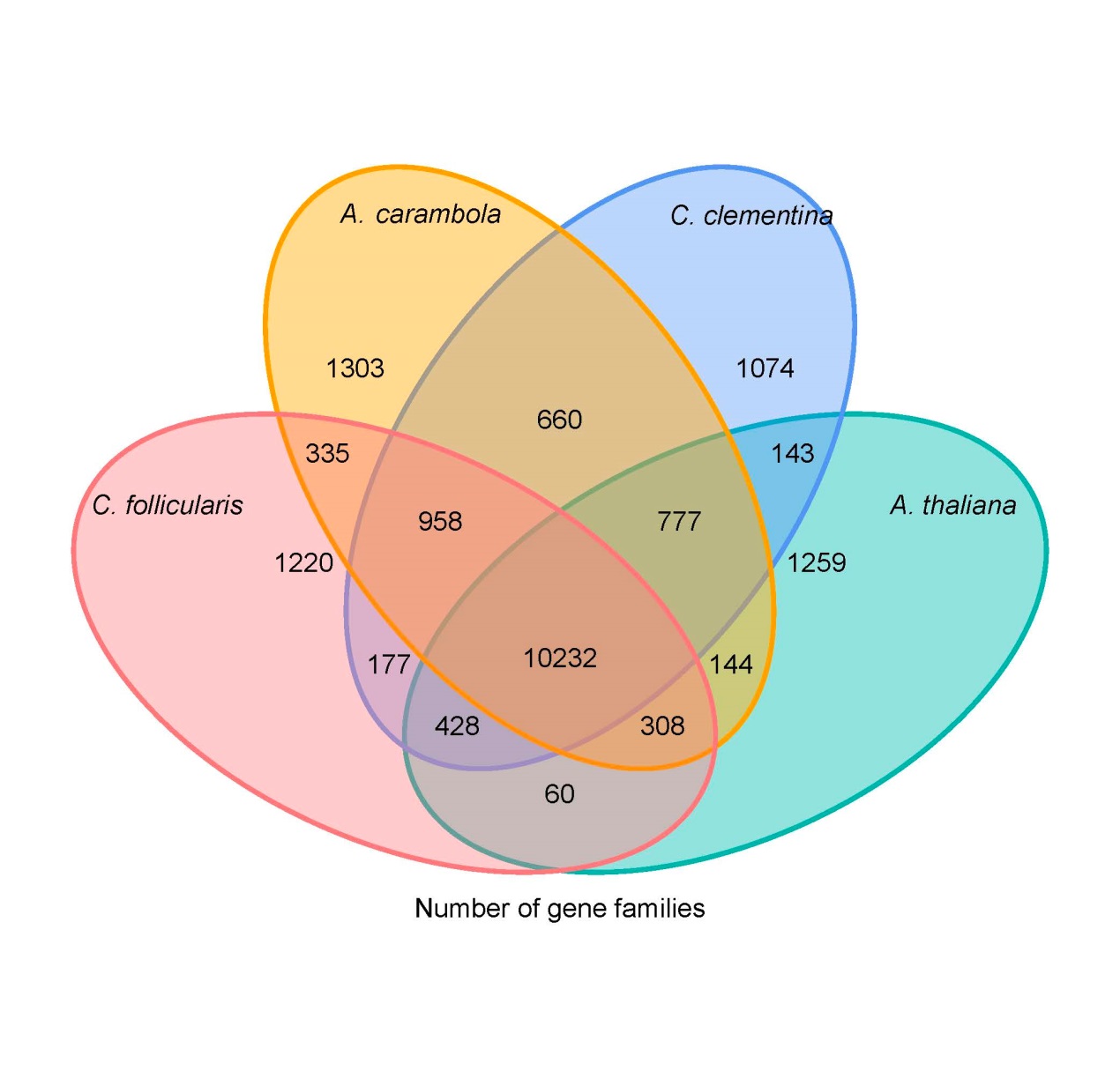


**Supplementary Figure 7. Venn diagram shows the number of orthologous genes families in *A. carambola*, *C. clementina*, *A. thaliana* and *C. follicularis*.**


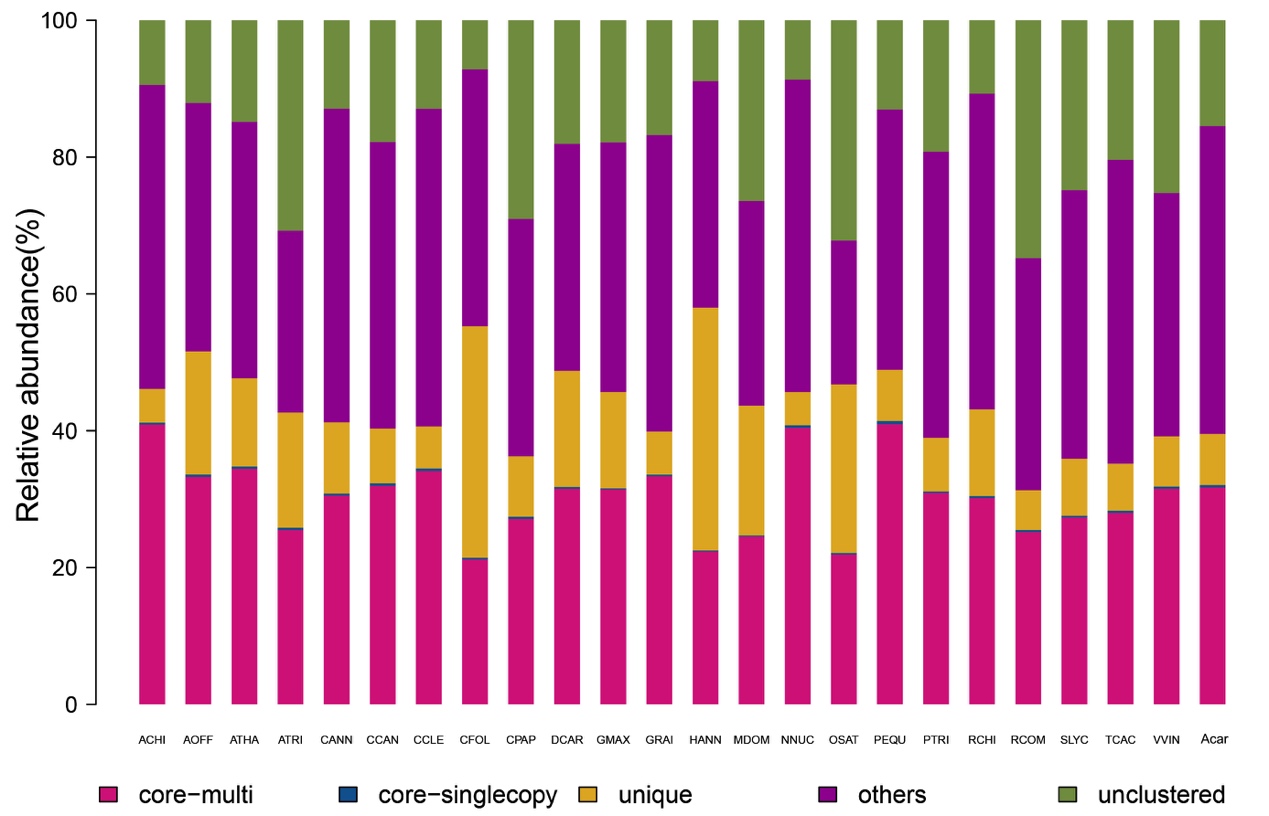


**Supplementary Figure 8. Orthologous genes in *A. carambola* and other species.**


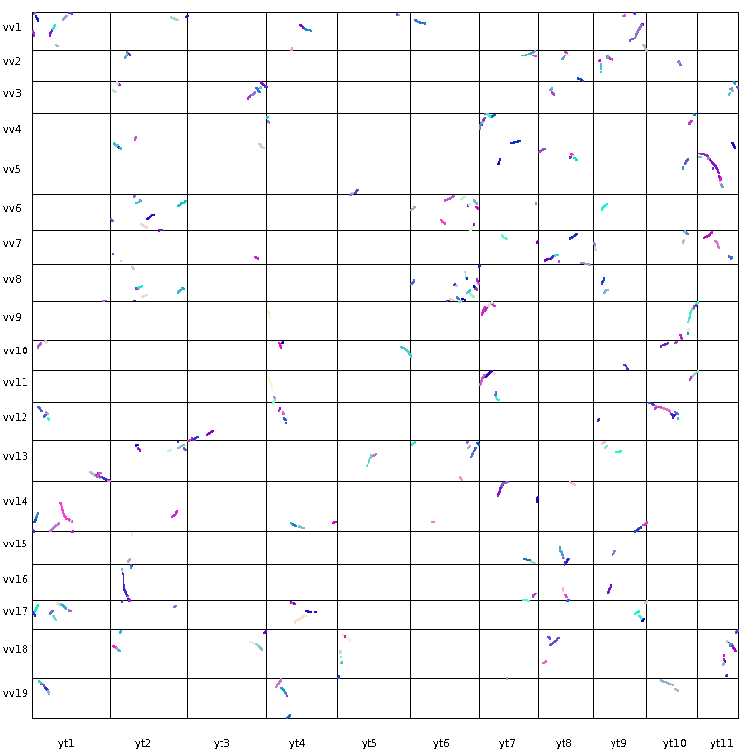


**Supplementary Figure 9. Collinear plot of *A. carambola* with *Vitis vinifera*.**


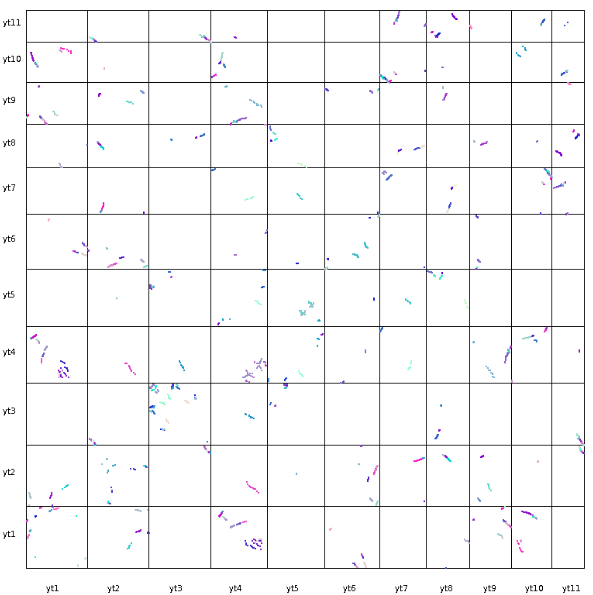


**Supplementary Figure 10. Collinear plot of *A. carambola*.**


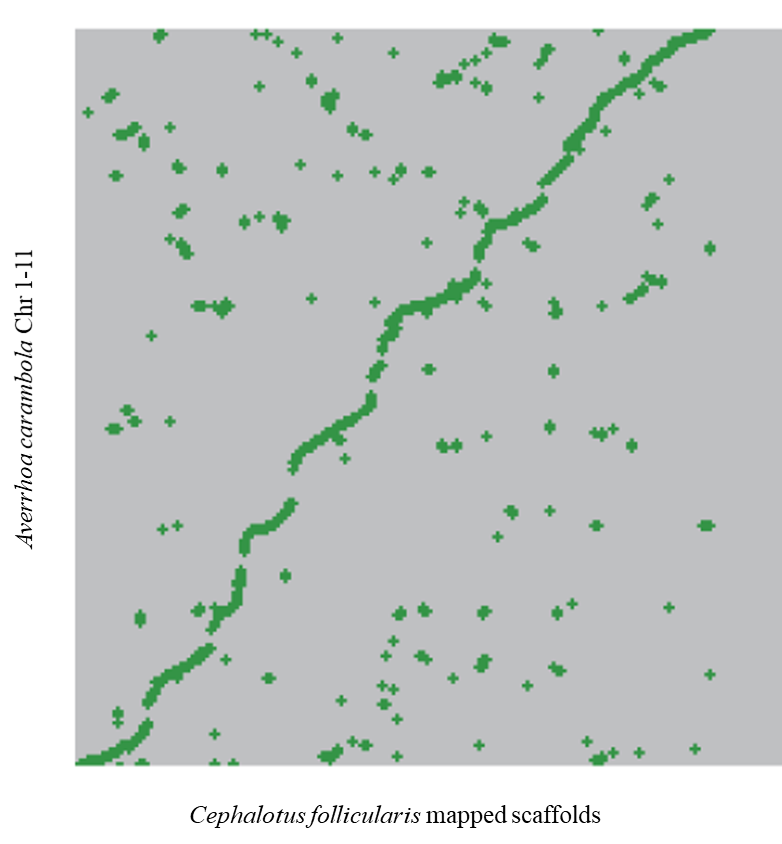


**Supplementary Figure 11. Genome comparison between *A. carambola* and *C. follicularis* genome.**


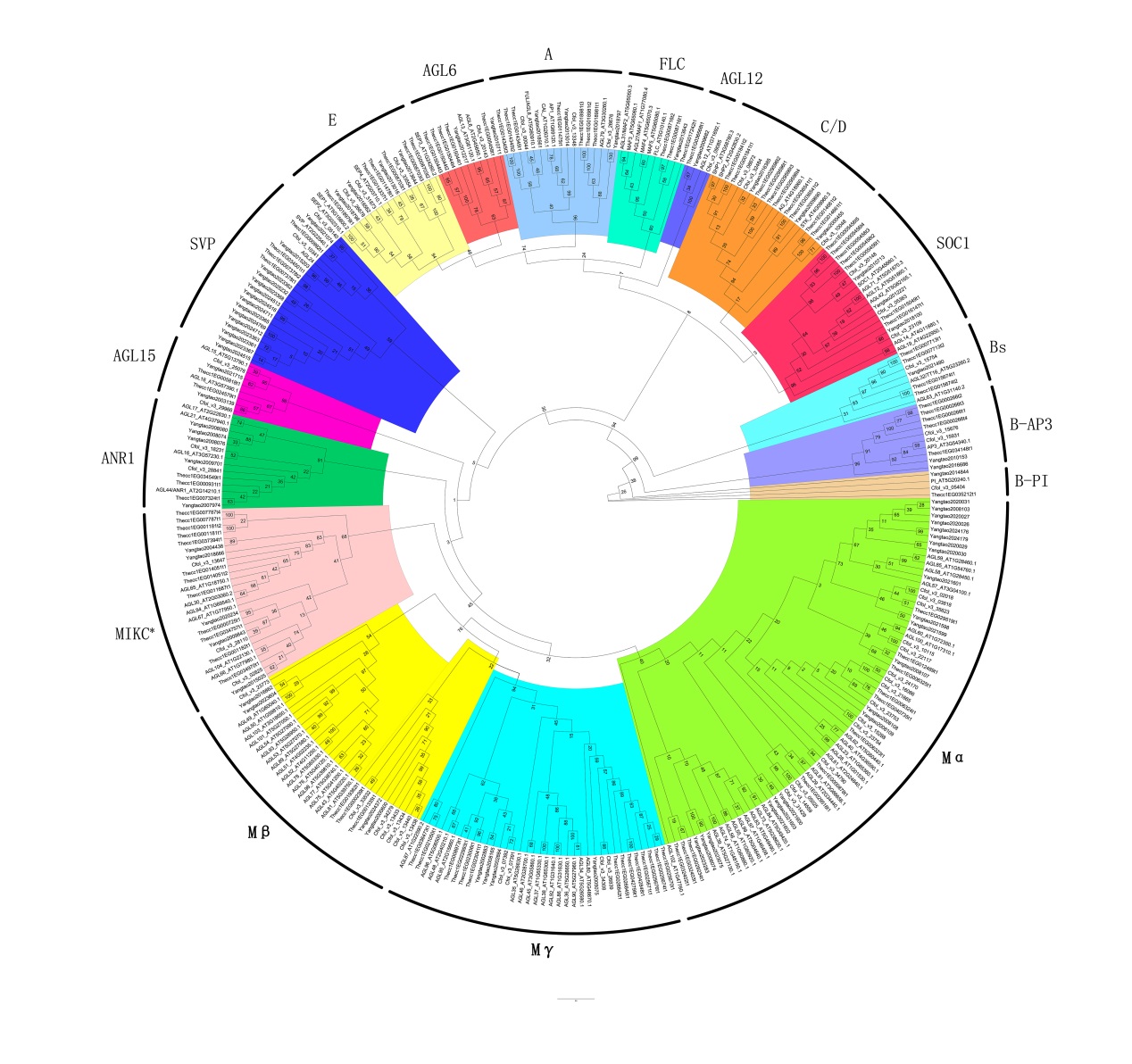


**Supplementary Figure 12. Phylogenetic tree showing the evolutionary relationship between MADS-box genes in the *A. carambola,*** ***C. follicularis*, *T. cacao* and *A. thaliana*.**

**
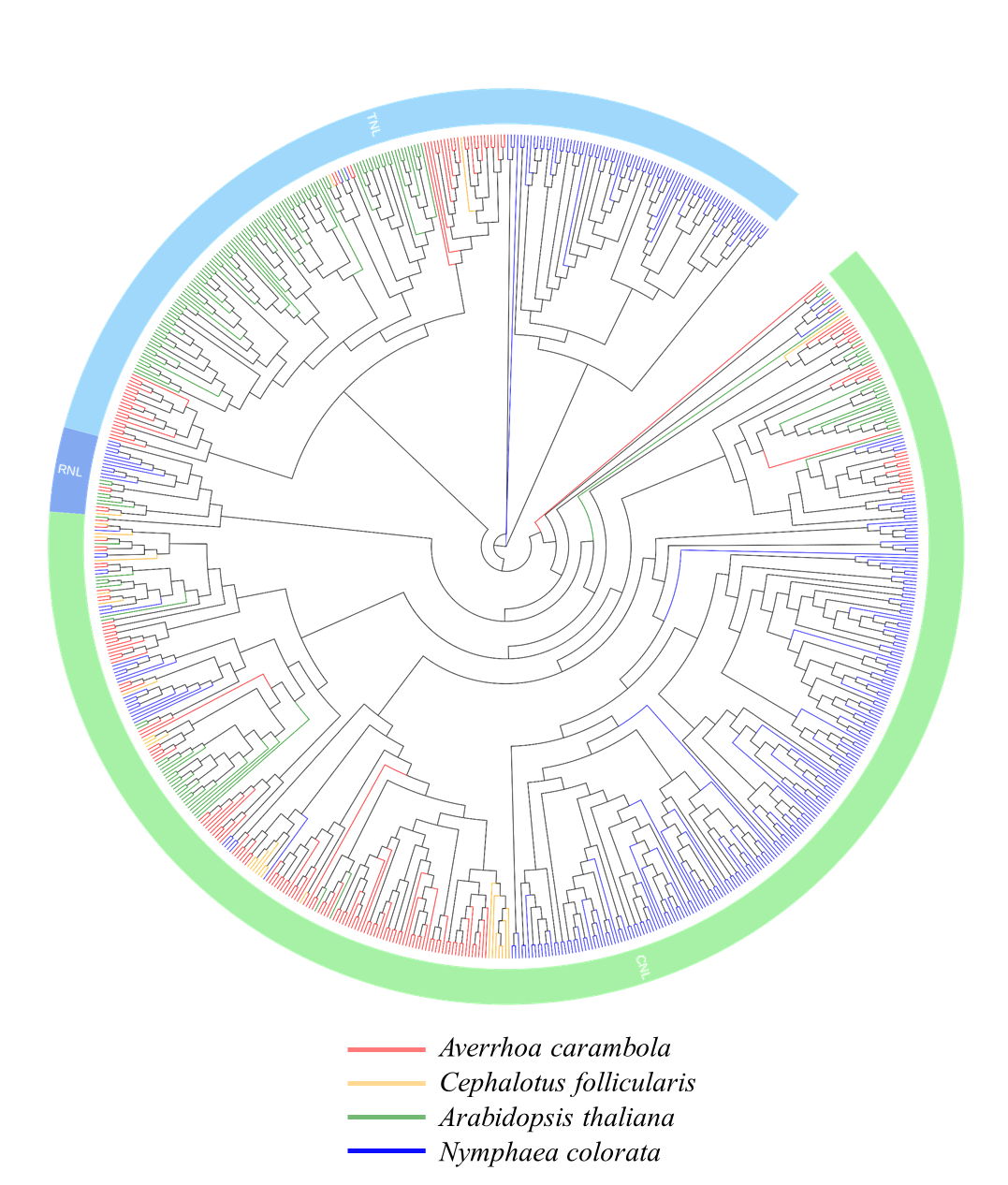
**

**Supplementary Figure 13. Phylogenetic tree showing the evolutionary relationship of *R* genes in the *A. carambola*, *C. follicularis*, *A. thaliana*, and *N. colorata.***


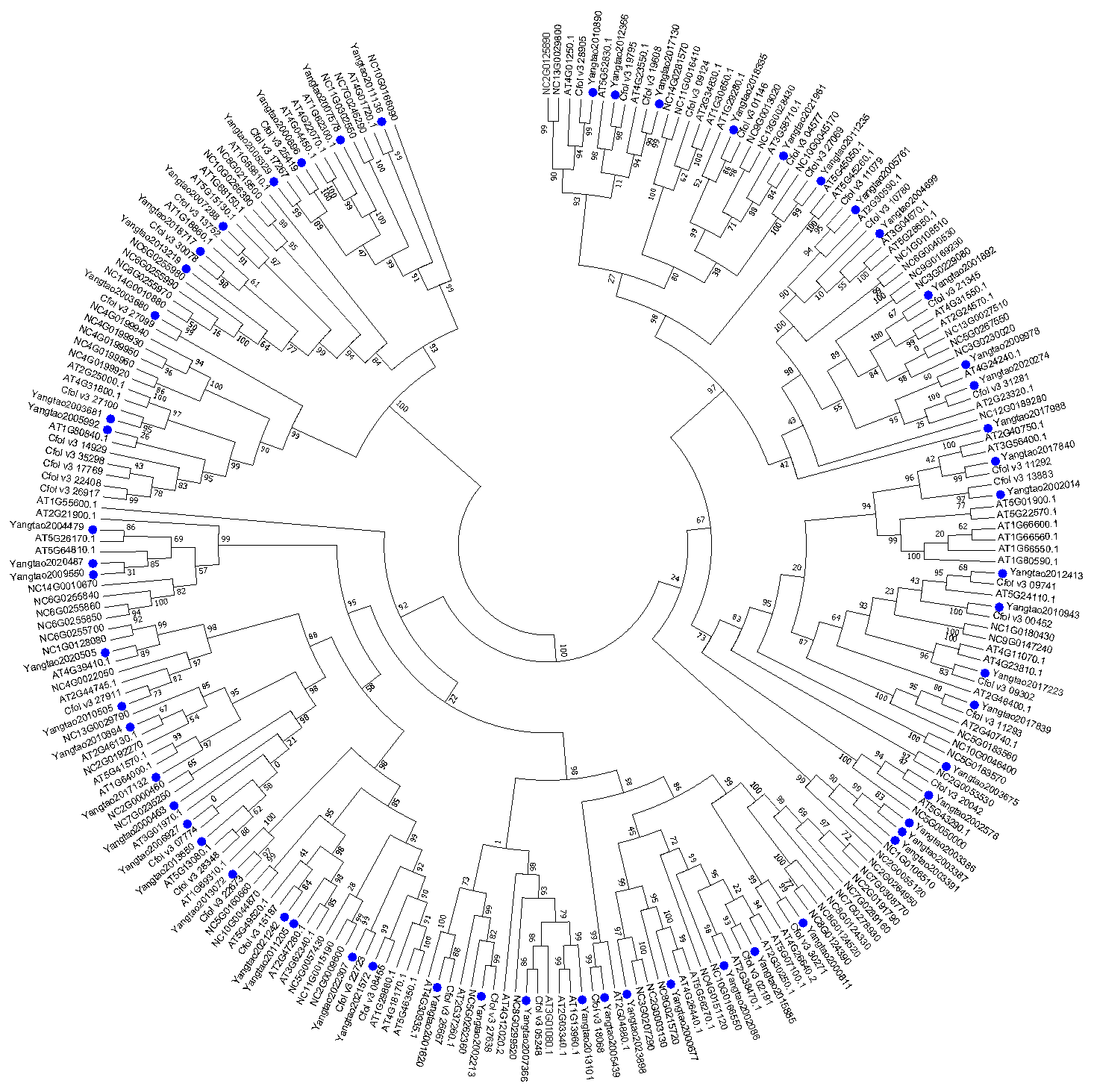


**Supplementary Figure 14. Phylogenetic tree showing the evolutionary relationship of WRKY genes in the *A. carambola*, *C. follicularis*, *A. thaliana*, and *N. colorata.***

Supplementary Tables

**Supplementary Table 1. The statistics of sequencing raw data from Illumina and Nanopore platforms.**

| **Platforms** | **Insert Size** | **Read_Length (N50)** | **Reads** | **Bases** |
| --- | --- | --- | --- | --- |
| Illumina Hiseq Xten | 400 bp | 150 bp | 873,370,026 | 131,005,503,900 bp |
| Oxford Nanopore | >20 kb | 28,990 bp | 3,863,525 | 52,332,657,343 bp |

**Supplementary Table 2. The assembled statistics of *A. carambola* genome.**

|  | *A. carambola* |
| --- | --- |
| 17-kmer (bp) | 392,185,678 |
| Assembled genome (bp) | 335,488,707 |
| Assemble genome / predicted genome | 90.31% |
| Contig N50 (bp) | 4,219,540 |
| BUSCO | C: 96.3% (S:94.2%, D:2.1%), F: 0.9%, M: 2.8%, n: 1375 |
| Chromosome | 11 |
| Chromosomal genome (bp) | 305,125,962 |
| Scaffold N50 (bp) | 31,246,569 |

**Supplementary Table 3. BUSCO assessment of the *A. carambola* genome.**

|  | Assembly | |
| --- | --- | --- |
|  | Proteins | Percentage |
| Complete Single-Copy BUSCOs | 1295 | 94.2 |
| Complete Duplicated BUSCOs | 29 | 2.1 |
| Fragmented BUSCOs | 13 | 0.9 |
| Missing BUSCOs | 38 | 2.8 |
| Total BUSCO groups searched | 1375 | 100.00 |

**Supplementary Table 4. The statistic result of Hi-C assembly.**

|  | Length(bp) | Number |
| --- | --- | --- |
| N50 | 31,246,569 | 5 |
| N90 | 21,943,723 | 10 |
| Total | 305,125,962 |  |
| Anchored rate | 90.88% |  |

**Supplementary Table 5. The length of chromosome by Hi-C assembly.**

| **ID** | **Length(bp)** |
| --- | --- |
| **Chr01** | 33,981,103 |
| **Chr02** | 32,937,330 |
| **Chr03** | 32,910,857 |
| **Chr04** | 31,438,085 |
| **Chr05** | 31,246,569 |
| **Chr06** | 29,477,223 |
| **Chr07** | 25,682,180 |
| **Chr08** | 24,317,693 |
| **Chr09** | 23,299,421 |
| **Chr10** | 21,943,723 |
| **Chr11** | 17,891,778 |
| **Total** | 305,125,962 |

**Supplementary Table 6. The statistic result of repeat sequence of *A. carambola*.**

| Type | Repeat Size(bp) | % of genome |
| --- | --- | --- |
| TRF | 10,139,049 | 2.97 |
| RepeatMasker | 52,865,175 | 15.50 |
| RepeatProteinMask | 48,122,103 | 14.11 |
| *De novo* | 149,012,221 | 43.70 |
| Total | 209,016,329 | 61.30 |

**Supplementary Table 7.** **The prediction of gene structures of the *A. carambola* genome.**

| **Gene set** | | **Protein coding gene number** | **Average gene length (bp)** | **Average CDS length (bp)** | **Average exon per gene** | **Average exon length (bp)** | **Average intron length (bp)** |
| --- | --- | --- | --- | --- | --- | --- | --- |
|  |  |  |  |  |  |  |  |
|  |  |  |  |  |  |  |  |
|  |  |  |  |  |  |  |  |
| De novo | AUGUSTUS | 22,972 | 2,843.16 | 1,232.89 | 5.58 | 221.02 | 351.74 |
|  | Genscan | 20,802 | 8,441.01 | 1,274.05 | 6.06 | 210.18 | 1,415.95 |
|  | GlimmerHMM | 34,020 | 7,864.04 | 898.47 | 4.22 | 212.68 | 2,160.19 |
| Homolog | *A. carambola* ‘Suanren’ | 23,902 | 3,433.85 | 1,215.46 | 4.97 | 244.65 | 559.04 |
|  | *A. thaliana* | 29,485 | 2,906.03 | 931.58 | 3.90 | 239.10 | 681.73 |
|  | *C. sinensis* | 25,737 | 2,929.76 | 935.58 | 4.11 | 227.90 | 642.22 |
|  | *G. max* | 28,340 | 2,723.76 | 926.84 | 3.91 | 237.01 | 617.36 |
|  | *R. rugosa* | 27,857 | 3,500.17 | 931.41 | 3.91 | 238.02 | 881.75 |
|  | *V. vinifera* | 28,527 | 3,087.83 | 945.26 | 3.92 | 241.41 | 734.86 |
| RNA-seq | | 21,316 | 4,070.44 | 1,186.59 | 4.99 | 237.96 | 395.01 |
| CEGMA | | 454 | 3,762.78 | 1,226.30 | 8.52 | 143.86 | 337.11 |
| MAKER | | 24,770 | 4,185.16 | 1,188.75 | 5.37 | 271.52 | 624.95 |
| Final set | | 25,419 | 3,207.68 | 1,154.00 | 4.92 | 240.03 | 506.20 |

**Supplementary Table 8. BUSCO assessment of gene prediction of the *A. carambola* genome.**

|  | Gene prediction | |
| --- | --- | --- |
|  | Proteins | Percentage |
| Complete Single-Copy BUSCOs | 1266 | 92.1 |
| Complete Duplicated BUSCOs | 37 | 2.7 |
| Fragmented BUSCOs | 33 | 2.4 |
| Missing BUSCOs | 39 | 2.8 |
| Total BUSCO groups searched | 1375 | 100.00 |

**Supplementary Table 9. Statistics on the annotation of Non-coding RNA of the *A. carambola* genome.**

| **Type** | | **Copy** | **Average length (bp)** | **Total length (bp)** | **% of genome** |
| --- | --- | --- | --- | --- | --- |
| miRNA | | 86 | 128.8256 | 11,079 | 0.003249 |
| tRNA | | 581 | 74.66954 | 43,383 | 0.012724 |
| rRNA | rRNA | 71 | 280.2958 | 19,901 | 0.005837 |
|  | 18S | 7 | 1,596.714 | 11,177 | 0.003278 |
|  | 28S | 13 | 189.4615 | 2,463 | 0.000722 |
|  | 5.8S | 7 | 163.7143 | 1,146 | 0.000336 |
|  | 5S | 44 | 116.25 | 5,115 | 0.0015 |
|  | snRNA | 212 | 117.3066 | 24,869 | 0.007294 |
| snRNA | CD-box | 130 | 102.9154 | 13,379 | 0.003924 |
|  | HACA-box | 16 | 131.3125 | 2,101 | 0.000616 |
|  | splicing | 66 | 142.2576 | 9,389 | 0.002754 |
|  | scaRNA | 0 | 0 | 0 | 0 |

**Supplementary Table 10. Statistic result of clustered gene families.**

| **Species** | **Genes** | **Unclustered genes** | **Clustered genes** | **Families** | **Unique families** | **Unique families**  **genes** | **Common families** | **Common**  **families genes** | **Single**  **copy** | **Average genes**  **per family** |
| --- | --- | --- | --- | --- | --- | --- | --- | --- | --- | --- |
| *A. carambola* | 25,419 | 3,932 | 21,487 | 14,717 | 504 | 1,898 | 5,408 | 8,153 | 93 | 1.46 |
| *A. chinensis* | 32,962 | 3,114 | 29,848 | 14,386 | 598 | 1,625 | 5,408 | 13,574 | 93 | 2.075 |
| *A. officinalis* | 26,005 | 3,144 | 22,861 | 12,109 | 735 | 4,679 | 5,408 | 8,739 | 93 | 1.888 |
| *A. thaliana* | 27,416 | 4,069 | 23,347 | 13,351 | 840 | 3,538 | 5,408 | 9,532 | 93 | 1.749 |
| *A. trichopoda* | 26,846 | 8,254 | 18,592 | 12,476 | 1,048 | 4,518 | 5,408 | 6,933 | 93 | 1.49 |
| *C. annuum* | 30,690 | 3,970 | 26,720 | 15,518 | 667 | 3,197 | 5,408 | 9,457 | 93 | 1.722 |
| *C. canephora* | 25,574 | 4,550 | 21,024 | 14,066 | 606 | 2,054 | 5,408 | 8,262 | 93 | 1.495 |
| *C. clementina* | 24,533 | 3,176 | 21,357 | 14,449 | 421 | 1,506 | 5,408 | 8,461 | 93 | 1.478 |
| *C. follicularisa* | 36,503 | 2,626 | 33,877 | 13,718 | 641 | 12,351 | 5,408 | 7,825 | 93 | 2.47 |
| *C. papaya* | 27,751 | 8,061 | 19,690 | 13,879 | 536 | 2,451 | 5,408 | 7,613 | 93 | 1.419 |
| *D. carota* | 32,113 | 5,807 | 26,306 | 14,062 | 1,323 | 5,449 | 5,408 | 10,210 | 93 | 1.871 |
| *G. max* | 56,044 | 9,996 | 46,048 | 16,104 | 2,358 | 7,892 | 5,408 | 17,699 | 93 | 2.859 |
| *G. raimondii* | 37,505 | 6,291 | 31,214 | 15,807 | 691 | 2,364 | 5,408 | 12,597 | 93 | 1.975 |
| *H. annuus* | 58,148 | 5,177 | 52,971 | 14,893 | 1,996 | 20,636 | 5,408 | 13,086 | 93 | 3.557 |
| *M. domestica* | 63,514 | 16,779 | 46,735 | 17,937 | 3,982 | 12,066 | 5,408 | 15,674 | 93 | 2.606 |
| *N. nucifera* | 23,884 | 2,070 | 21,814 | 13,188 | 342 | 1,158 | 5,408 | 9,747 | 93 | 1.654 |
| *O. sativa* | 42,189 | 13,587 | 28,602 | 13,651 | 2,122 | 10,397 | 5,408 | 9,338 | 93 | 2.095 |
| *P.equestris* | 19,510 | 2,543 | 16,967 | 11,589 | 445 | 1,460 | 5,408 | 8,083 | 93 | 1.464 |
| *P. trichocarpa* | 41,335 | 7,945 | 33,390 | 15,554 | 929 | 3,238 | 5,408 | 12,871 | 93 | 2.147 |
| *R. chinensis* | 30,138 | 3,231 | 26,907 | 14,941 | 842 | 3,814 | 5,408 | 9,180 | 93 | 1.801 |
| *R. communis* | 31,221 | 10,851 | 20,370 | 15,010 | 664 | 1,820 | 5,408 | 7,957 | 93 | 1.357 |
| *S. lycopersicum* | 34,725 | 8,625 | 26,100 | 15,957 | 726 | 2,893 | 5,408 | 9,577 | 93 | 1.636 |
| *T. cacao* | 29,452 | 6,006 | 23,446 | 15,582 | 441 | 2,023 | 5,408 | 8,339 | 93 | 1.505 |
| *V. vinifera* | 26,346 | 6,652 | 19,694 | 13,536 | 631 | 1,928 | 5,408 | 8,393 | 93 | 1.455 |

**Supplementary Table 11. Statistics on the annotation of the *A. carambola* genome.**

|  | | Number | Percent (%) |
| --- | --- | --- | --- |
| Total | | 25,419 | 100.00 |
| Annotated | InterPro | 24,779 | 97.4822 |
|  | GO | 22,775 | 89.59833 |
|  | KEGG | 13,899 | 54.67957 |
|  | Swissprot | 16,431 | 64.64062 |
|  | TrEMBL | 24,250 | 95.40108 |
| Unannotated | | 241 | 0.94811 |

**Supplementary Table 12. GO enrichment of significant expansion of *A. carambola* gene families.**

| GO ID | GO Term | GO Class | Pvalue | AdjustedPv | x1 | x2 | n | N | GOlevl |
| --- | --- | --- | --- | --- | --- | --- | --- | --- | --- |
| GO:0050660 | flavin adenine dinucleotide binding | MF | 3.56E-72 | 2.79E-69 | 55 | 140 | 274 | 25419 | 5 |
| GO:0008762 | UDP-N-acetylmuramate dehydrogenase activity | MF | 2.92E-64 | 2.29E-61 | 40 | 62 | 274 | 25419 | 6 |
| GO:0016491 | oxidoreductase activity | MF | 1.90E-26 | 1.48E-23 | 96 | 2780 | 274 | 25419 | 3 |
| GO:0055114 | oxidation-reduction process | BP | 2.46E-26 | 1.93E-23 | 93 | 2625 | 274 | 25419 | 4 |
| GO:0003824 | catalytic activity | MF | 2.34E-25 | 1.84E-22 | 189 | 9684 | 274 | 25419 | 2 |
| GO:0008152 | metabolic process | BP | 7.15E-23 | 5.60E-20 | 203 | 11428 | 274 | 25419 | 2 |
| GO:0016758 | transferase activity, transferring hexosyl groups | MF | 2.48E-22 | 1.94E-19 | 35 | 381 | 274 | 25419 | 5 |
| GO:0044710 | single-organism metabolic process | BP | 6.78E-19 | 5.31E-16 | 111 | 4519 | 274 | 25419 | 3 |
| GO:0036094 | small molecule binding | MF | 3.80E-18 | 2.98E-15 | 93 | 3431 | 274 | 25419 | 3 |
| GO:0000166 | nucleotide binding | MF | 1.49E-17 | 1.17E-14 | 91 | 3376 | 274 | 25419 | 4 |
| GO:0050661 | NADP binding | MF | 2.51E-16 | 1.97E-13 | 14 | 52 | 274 | 25419 | 5 |
| GO:0016740 | transferase activity | MF | 1.23E-13 | 9.60E-11 | 79 | 3111 | 274 | 25419 | 3 |
| GO:1901363 | heterocyclic compound binding | MF | 1.95E-12 | 1.52E-09 | 138 | 7679 | 274 | 25419 | 3 |
| GO:0097159 | organic cyclic compound binding | MF | 2.03E-12 | 1.59E-09 | 138 | 7683 | 274 | 25419 | 3 |
| GO:0043168 | anion binding | MF | 1.83E-11 | 1.43E-08 | 78 | 3360 | 274 | 25419 | 4 |
| GO:0003887 | DNA-directed DNA polymerase activity | MF | 1.27E-10 | 9.91E-08 | 12 | 85 | 274 | 25419 | 7 |
| GO:0016705 | oxidoreductase activity, acting on paired donors, with incorporation or reduction of molecular oxygen | MF | 5.37E-09 | 4.21E-06 | 34 | 1017 | 274 | 25419 | 4 |
| GO:0016779 | nucleotidyltransferase activity | MF | 1.20E-08 | 9.43E-06 | 20 | 393 | 274 | 25419 | 5 |
| GO:0016884 | carbon-nitrogen ligase activity, with glutamine as amido-N-donor | MF | 3.58E-08 | 2.80E-05 | 6 | 19 | 274 | 25419 | 5 |
| GO:0008146 | sulfotransferase activity | MF | 6.57E-08 | 5.14E-05 | 7 | 34 | 274 | 25419 | 5 |
| … | … | … | … | … | … | … | … | … | … |

**Supplementary Table 13. KEGG pathway enrichment of significant expansion of *A. carambola* gene families.**

| MapID | MapTitle | Pvalue | AdjustedPv | x | y | n | N |
| --- | --- | --- | --- | --- | --- | --- | --- |
| map00908 | Zeatin biosynthesis | 8.71E-24 | 2.61E-22 | 26 | 152 | 274 | 25419 |
| map00460 | Cyanoamino acid metabolism | 6.64E-21 | 1.99E-19 | 22 | 121 | 274 | 25419 |
| map01110 | Biosynthesis of secondary metabolites | 1.16E-13 | 3.47E-12 | 55 | 1686 | 274 | 25419 |
| map00380 | Tryptophan metabolism | 1.10E-10 | 3.29E-09 | 12 | 84 | 274 | 25419 |
| map04626 | Plant-pathogen interaction | 5.64E-09 | 1.69E-07 | 30 | 817 | 274 | 25419 |
| map01100 | Metabolic pathways | 1.64E-08 | 4.92E-07 | 65 | 2972 | 274 | 25419 |
| map04712 | Circadian rhythm - plant | 1.62E-07 | 4.85E-06 | 11 | 129 | 274 | 25419 |
| map00941 | Flavonoid biosynthesis | 1.75E-07 | 5.25E-06 | 11 | 130 | 274 | 25419 |
| map00966 | Glucosinolate biosynthesis | 3.06E-07 | 9.18E-06 | 7 | 42 | 274 | 25419 |
| map00190 | Oxidative phosphorylation | 2.27E-05 | 0.00068 | 10 | 176 | 274 | 25419 |
| map00620 | Pyruvate metabolism | 0.000129 | 0.003856 | 7 | 103 | 274 | 25419 |
| map00970 | Aminoacyl-tRNA biosynthesis | 0.000199 | 0.005976 | 6 | 78 | 274 | 25419 |
| map00730 | Thiamine metabolism | 0.000625 | 0.018758 | 3 | 16 | 274 | 25419 |
| map00195 | Photosynthesis | 0.000646 | 0.019394 | 6 | 97 | 274 | 25419 |
| map00908 | Zeatin biosynthesis | 8.71E-24 | 2.61E-22 | 26 | 152 | 274 | 25419 |
| … | … | … | … | … | … | … | … |

**Supplementary Table 14. GO enrichment of significant contraction of *A. carambola* gene families.**

| GO ID | GO Term | GO Class | Pvalue | AdjustedPv | x1 | x2 | n | N | GOlevl |
| --- | --- | --- | --- | --- | --- | --- | --- | --- | --- |
| GO:0042626 | ATPase activity, coupled to transmembrane movement of substances | MF | 1.86E-14 | 8.15E-12 | 13 | 244 | 61 | 25419 | 5 |
| GO:0003824 | catalytic activity | MF | 3.88E-14 | 1.70E-11 | 52 | 9684 | 61 | 25419 | 2 |
| GO:0043167 | ion binding | MF | 8.19E-13 | 3.59E-10 | 41 | 6051 | 61 | 25419 | 3 |
| GO:0005524 | ATP binding | MF | 9.84E-13 | 4.31E-10 | 27 | 2381 | 61 | 25419 | 6 |
| GO:0097367 | carbohydrate derivative binding | MF | 4.92E-11 | 2.15E-08 | 28 | 3046 | 61 | 25419 | 3 |
| GO:0004713 | protein tyrosine kinase activity | MF | 8.72E-09 | 3.82E-06 | 14 | 839 | 61 | 25419 | 7 |
| GO:1901363 | heterocyclic compound binding | MF | 5.41E-08 | 2.37E-05 | 39 | 7679 | 61 | 25419 | 3 |
| GO:0097159 | organic cyclic compound binding | MF | 5.49E-08 | 2.41E-05 | 39 | 7683 | 61 | 25419 | 3 |
| GO:0022804 | active transmembrane transporter activity | MF | 1.95E-07 | 8.55E-05 | 14 | 1078 | 61 | 25419 | 4 |
| GO:0005488 | binding | MF | 2.46E-07 | 0.000108 | 49 | 12263 | 61 | 25419 | 2 |
| GO:0006468 | protein phosphorylation | BP | 6.06E-07 | 0.000265 | 14 | 1184 | 61 | 25419 | 7 |
| GO:0055085 | transmembrane transport | BP | 1.12E-06 | 0.000489 | 13 | 1062 | 61 | 25419 | 4 |
| GO:0009055 | electron carrier activity | MF | 2.26E-06 | 0.000988 | 10 | 627 | 61 | 25419 | 2 |
| GO:0005506 | iron ion binding | MF | 4.17E-06 | 0.001827 | 10 | 672 | 61 | 25419 | 7 |
| GO:0020037 | heme binding | MF | 1.35E-05 | 0.005905 | 11 | 945 | 61 | 25419 | 5 |
| GO:0016772 | transferase activity, transferring phosphorus-containing groups | MF | 2.62E-05 | 0.011491 | 15 | 1870 | 61 | 25419 | 4 |
| GO:0016705 | oxidoreductase activity, acting on paired donors, with incorporation or reduction of molecular oxygen | MF | 2.66E-05 | 0.011645 | 11 | 1017 | 61 | 25419 | 4 |
| GO:0016787 | hydrolase activity | MF | 3.68E-05 | 0.01614 | 22 | 3791 | 61 | 25419 | 3 |

**Supplementary Table 15. KEGG pathway enrichment of significant contraction of *A. carambola* gene families.**

| MapID | MapTitle | Pvalue | AdjustedPv | x | y | n | N |
| --- | --- | --- | --- | --- | --- | --- | --- |
| map00903 | Limonene and pinene degradation | 4.30E-11 | 8.16E-10 | 9 | 138 | 61 | 25419 |
| map00945 | Stilbenoid, diarylheptanoid and gingerol biosynthesis | 8.20E-10 | 1.56E-08 | 9 | 192 | 61 | 25419 |
| map01110 | Biosynthesis of secondary metabolites | 1.09E-09 | 2.07E-08 | 20 | 1686 | 61 | 25419 |
| map00460 | Cyanoamino acid metabolism | 1.08E-05 | 0.000205 | 5 | 121 | 61 | 25419 |
| map00514 | Other types of O-glycan biosynthesis | 1.24E-05 | 0.000236 | 3 | 19 | 61 | 25419 |
| map00909 | Sesquiterpenoid and triterpenoid biosynthesis | 3.69E-05 | 0.000701 | 3 | 27 | 61 | 25419 |
| map01100 | Metabolic pathways | 0.000144 | 0.002735 | 18 | 2972 | 61 | 25419 |
| map00940 | Phenylpropanoid biosynthesis | 0.000461 | 0.008762 | 5 | 268 | 61 | 25419 |
| map04626 | Plant-pathogen interaction | 0.000719 | 0.013658 | 8 | 817 | 61 | 25419 |
| map00500 | Starch and sucrose metabolism | 0.001503 | 0.028554 | 5 | 349 | 61 | 25419 |

**Supplementary Table 16. GO enrichment of unique genes of *A. carambola.***

| GO ID | GO Term | GO Class | Pvalue | AdjustedPv | x1 | x2 | n | N | GOlevl |
| --- | --- | --- | --- | --- | --- | --- | --- | --- | --- |
| GO:0015074 | DNA integration | BP | 3.17E-19 | 5.39E-16 | 38 | 93 | 1898 | 25419 | 6 |
| GO:0033897 | ribonuclease T2 activity | MF | 7.81E-15 | 1.33E-11 | 15 | 18 | 1898 | 25419 | 9 |
| GO:0046983 | protein dimerization activity | MF | 1.83E-11 | 3.11E-08 | 66 | 366 | 1898 | 25419 | 4 |
| GO:0008234 | cysteine-type peptidase activity | MF | 1.84E-06 | 0.003118 | 27 | 134 | 1898 | 25419 | 6 |
| GO:0016844 | strictosidine synthase activity | MF | 3.28E-06 | 0.005573 | 9 | 19 | 1898 | 25419 | 6 |
| GO:0006259 | DNA metabolic process | BP | 4.79E-06 | 0.00812 | 157 | 1484 | 1898 | 25419 | 5 |
| GO:0004521 | endoribonuclease activity | MF | 7.46E-06 | 0.012667 | 23 | 112 | 1898 | 25419 | 7 |
| GO:0004540 | ribonuclease activity | MF | 8.93E-06 | 0.015148 | 24 | 121 | 1898 | 25419 | 6 |
| GO:0004197 | cysteine-type endopeptidase activity | MF | 1.15E-05 | 0.019575 | 14 | 50 | 1898 | 25419 | 7 |
| GO:0008171 | O-methyltransferase activity | MF | 2.53E-05 | 0.042873 | 15 | 60 | 1898 | 25419 | 6 |
| GO:0005332 | gamma-aminobutyric acid:sodium symporter activity | MF | 2.75E-05 | 0.046639 | 7 | 14 | 1898 | 25419 | 8 |
| GO:0003964 | RNA-directed DNA polymerase activity | MF | 2.81E-05 | 0.047759 | 22 | 113 | 1898 | 25419 | 7 |

**Supplementary Table 17. KEGG pathway enrichment of unique genes of *A. carambola.***

| MapID | MapTitle | Pvalue | AdjustedPv | x | y | n | N |
| --- | --- | --- | --- | --- | --- | --- | --- |
| map03020 | RNA polymerase | 1.52E-08 | 9.73E-07 | 42 | 220 | 1898 | 25419 |
| map00901 | Indole alkaloid biosynthesis | 1.05E-06 | 6.75E-05 | 11 | 26 | 1898 | 25419 |
| map03018 | RNA degradation | 3.90E-06 | 0.00025 | 40 | 250 | 1898 | 25419 |
| map00071 | Fatty acid metabolism | 1.11E-05 | 0.000712 | 18 | 77 | 1898 | 25419 |
| map03008 | Ribosome biogenesis in eukaryotes | 6.44E-05 | 0.004123 | 35 | 234 | 1898 | 25419 |
| map00350 | Tyrosine metabolism | 0.000137 | 0.008742 | 18 | 92 | 1898 | 25419 |
| map00402 | Benzoxazinoid biosynthesis | 0.000325 | 0.020805 | 8 | 25 | 1898 | 25419 |
| map00240 | Pyrimidine metabolism | 0.000679 | 0.043438 | 42 | 335 | 1898 | 25419 |
